# Single-Cell dissection of fibrodysplasia ossificans progressiva identifies SPP1 as a mediator of macrophage–fibroadipogenic progenitors crosstalk

**DOI:** 10.1007/s00018-026-06320-z

**Published:** 2026-07-02

**Authors:** Riccardo Gamberale, Mauro Bergamaschi, Anna Sofia Tascini, Cristina D’Orlando, Michela Signo, Antonello Spinelli, Raffaella Meneveri, Renata Bocciardi, Bénédicte Chazaud, Emanuele Azzoni, Silvia Brunelli

**Affiliations:** 1 School of Medicine and Surgery, University of Milano-Bicocca, Monza, Italy; 2 Center for Omics Sciences, IRCCS Ospedale San Raffaele, Milan, Italy; 3 Preclinical Imaging Facility, IRCCS Ospedale San Raffaele, Milan, Italy; 4 DINOGMI, University of Genoa, Genoa, Italy; 5 IRCCS Istituto Giannina Gaslini, Genoa, Italy; 6 Institut NeuroMyoGène, Physiopathologie Et Génétique du Neurone Et du Muscle, UMR CNRS 5261, INSERM U1315, Université Claude Bernard, Lyon 1, Lyon, France; 7 Fondazione IRCCS San Gerardo Dei Tintori, Monza, Italy

**Keywords:** Heterotopic ossification, Fibrodysplasia ossificans progressiva, Macrophages, SPP1, Single-cell RNA sequencing

## Abstract

Fibrodysplasia Ossificans Progressiva (FOP) is a rare genetic disorder caused by gain-of-function mutations in ACVR1/ALK2, leading to progressive heterotopic ossification (HO) through endochondral bone formation. Inflammatory flare-ups often precede new ossification events, but the cellular and molecular mechanisms linking immune responses to progenitor cell fate remain incompletely understood. Here, using a tamoxifen-inducible Acvr1R206H mouse model of FOP and a reproducible muscle injury protocol, we combined single-cell RNA sequencing with *in vitro *assays to dissect early events during lesion formation. We identified an expansion of macrophages (MPs) and fibro-adipogenic progenitors (FAPs) in FOP mice, with both populations exhibiting inflammatory and osteochondrogenic transcriptional signatures. Cell–cell interaction analysis revealed a self-reinforcing network of cytokine signaling among MPs and a prominent MP–FAP communication axis centred on SPP1. Functional studies confirmed that SPP1 enhanced FAP osteogenic differentiation and that its inhibition partially reversed this phenotype* in vitro* and attenuated HO in *vivo*. Our findings highlight the critical role of inflammatory MPs in shaping the fate of resident stromal mesenchymal progenitors (e.g. FAP) and suggest that early immune–stromal interactions set the stage for HO. Targeting this immune–mesenchymal crosstalk may represent a potential complementary strategy for preventing or mitigating disease progression in FOP.

**Supplementary Information:**

The online version contains supplementary material available at https://doi.org/10.1007/s00018-026-06320-z.

## Introduction

Heterotopic ossification (HO) refers to the pathological formation of mature bone in soft tissues such as skeletal muscle, fascia, and tendons. While acquired forms of HO are often associated with trauma, burns, or surgical procedures, its most severe manifestation is observed in Fibrodysplasia Ossificans Progressiva (FOP) a rare, autosomal dominant disorder characterized by progressive and irreversible episodes of soft tissue ossification that severely impair mobility and shorten life expectancy [1, 2]. The genetic basis of FOP has been traced to activating mutations in the *ACVR1* gene, which encodes the type I receptor ALK2 for bone morphogenetic proteins (BMPs). The recurrent R206H mutation—found in ~ 97% of patients—renders ALK2 hyperactive and causes aberrant activation of mutant ACVR1/ALK2 signaling by Activin A, thereby promoting ectopic chondro-osteogenic differentiation and endochondral ossification in response to inflammation or minor injury [3–5].

Clinically, FOP progresses through episodic inflammatory flare-ups that are often unpredictable and may be spontaneous or trauma-induced. These flare-ups initiate local swelling, tissue inflammation, and ultimately lead to the replacement of soft tissue with bone. However, disease progression is not always linked to overt inflammatory episodes: “creeping” ossification has been reported even in the absence of visible flare-ups, underscoring the complexity of the mechanisms involved [6, 7].

FOP presents a profound unmet medical need due to its extreme rarity, debilitating nature, and lack of effective treatments. Several therapeutic strategies are under investigation, including small-molecule inhibitors of ALK2 (e.g., Saracatinib, BLU-782), retinoic acid receptor agonists (e.g., Palovarotene), and neutralizing antibodies against Activin A (e.g., Garetosmab) [8–10]. Although promising, these approaches face significant challenges, including serious side effects and the need to start early in infancy to prevent the HO leading to cumulative disability. This underscores the urgent need for novel, comprehensive strategies to address FOP, including multi-targeted strategies that go beyond single-pathway inhibition.

In recent years, the immune system has emerged as a pivotal modulator of HO. Both acquired and genetic models of the disease have revealed that immune cells, especially macrophages (MPs), accumulate in early lesions where they can produce a rich milieu of cytokines and growth factors, thus influencing tissue remodeling and ossification [11–13]. MPs during tissue remodelling exhibit a high degree of plasticity and may adopt either pro- or anti-inflammatory phenotypes depending on local cues [14, 15]. This functional heterogeneity makes them key regulators of the tissue environment during injury and repair. Studies have shown that MP depletion can suppress or delay HO, while pharmacological immune modulation—such as IL-1 inhibition—has yielded encouraging clinical responses in selected FOP patients [16].

Despite these findings, our understanding of how MPs interact with other cell types to initiate or amplify ossification remains incomplete. One cell population of growing interest is the fibro-adipogenic progenitors (FAPs)—mesenchymal stromal cells residing in skeletal muscle. FAPs are known to support regeneration by modulating the extracellular matrix and promoting muscle stem cell differentiation, but they can also adopt fibrogenic or osteogenic fates and have been found to contribute to endochondral bone repair upon fracture [17]. Recent evidence has demonstrated that FAPs are the primary osteochondral progenitors in FOP lesions, and that their differentiation is shaped by the inflammatory microenvironment [18, 19]. However, the specific molecular dialogues between MPs and FAPs that drive this transition remain poorly defined.

To address this issue, we combined a tamoxifen-inducible Acvr1R206H mouse model of FOP with single-cell RNA sequencing, bioinformatic analysis and *in vitr*o/*in vivo* functional assays. Our goal was to characterize the cellular and molecular dynamics of the early inflammatory phase following muscle injury, with a particular focus on the crosstalk between MPs and FAPs. By mapping cell–cell communication networks and functionally assessing MP-derived factors, we provide new insights into how the immune compartment contributes to the initiation of heterotopic ossification and identify Secreted Phosphoprotein-1 (SPP1) as a prominent component of the MP–FAP communication network.

Our findings reveal an altered MP-landscape and identify key inflammatory cues capable of reprogramming FAPs toward an osteogenic fate—highlighting signalling pathways that may contribute to pathological ossification and represent potential therapeutic targets in FOP.

## Materials and methods

### Animal model

We used the homozygous Acvr1^R206Hlox^ Gt(ROSA26)^SorCreERT2^ mouse model (C57BL/6 background), for the purpose of this work. This mouse model was kindly provided by the International Fibrodysplasia Ossificans Progressiva Association (IFOPA) and generated thanks to a collaboration with Daniel Perrien (Emory University School of Medicine) (https://www.ifopa.org/fop_mouse_model_2020). Mice were kept under controlled conditions (12 h light/dark cycle and room temperature at 22 °C) and had free access to tap water and standard mice chow.

Acvr1^R206Hlox^ Gt(ROSA26)^SorCreERT2^ mice were housed in the SPF facility at San Raffaele Scientific Institute (Milan, Italy) and treated with the approval of the Institutional Animal Care and Use Committee (IACUC 1325 Ministry of Health Authorization n. 575/2024-PR). Mice were randomly assigned to experimental groups (at least 3 animals/group/timepoint).

Both male and female mice were used in a randomized manner across experimental groups. No evident sex-dependent differences were observed in the i*n vivo* or *in vitro* phenotypes analyzed under the experimental conditions employed.

### In vivo ossification protocol

Cre activation in Acvr1^R206Hlox^ Gt(ROSA26)^SorCreERT2^ mice was induced with tamoxifen diet (TAM400/CreER, Envigo) for 10 days to generate Acvr1^R206H^Gt(ROSA26)^SorCreERT2^ mice (FOP mice) and recombination was tested by PCR. At 8 weeks of age pinch injury was performed by compressing the gastrocnemius muscle using a pair of tweezers for 15 s, leaving a space of roughly 1 mm between the ends of the tweezers. After all the experimental procedures, mice were euthanized and dissected for the collection of the injured muscles for further procedures.

### Depletion of circulating phagocytes

At the age of 8 weeks, FOP mice were intravenously injected with 200 μl liposomes containing Clodronate (CLL) or 200 μl PBS liposomes as control (SHAM). CLL and SHAM injections were performed one day before and two days after pinch injury. CLL and SHAM liposomes were purchased from: http://www.clodronateliposomes.org/ashwindigital.asp?docid=26.

To confirm an effective reduction of circulating monocytes in CLL vs SHAM treated mice, blood was collected 4 days after pinch injury and processed for FACS analysis. Briefly, samples were stained and analysed in CMF (Calcium/Magnesium-Free PBS, 10% FBS (Gibco, 10,270–106), 5% Pen/Strep (Gibco, 15,140–122), 2 mM EDTA), incubated for 15 min with 0.5 mg/mL Fc block (1:500) (BD Biosciences, Franklin Lakes, NJ, USA) and labelled with a combination of PE-conjugated rat anti-CD45 (1:400) (BD Biosciences), PE-Cy7-conjugated rat anti-CD11b (1:200) (BD Biosciences) and APC-conjugated rat anti-F4/80 (1:200) (BD Biosciences). Appropriate fluorescence gating parameters were established with compensation beads (BD Biosciences), unstained, and fluorescence-minus-one (FMO) staining. In all the samples, doublets were gated out using pulsegeometry gates (FSC-H *versus* FSC-A and SSC-H *versus* SSC-A), whereas dead cells were excluded using Hoechst 33,258 (Hellobio, Bristol, UK). Suspensions were analyzed using a Fortessa X-20 analyzer (BD Biosciences) and FlowJo software v10 (FlowJo/BD, Ashland, OR, USA).

### *In vivo *micro computed tomography imaging (µCT)

Micro computed tomography (μCT) was performed at day 14 and 21 to monitor HO after muscle injury. *In vivo* µCT imaging was performed using the IVIS SpectrumCT Pre-clinical in Vivo Imaging System (Perkin-Elmer, Waltham, MA, USA). µCT images were acquired without any contrast medium, with the following parameters: x-ray tube voltage = 50 kV, tube current = 1 mA, x-ray focal spot size = 50 μm. The µCT images calibrated in Hounsfield unit (HU) were reconstructed with a voxel size of 75 μm^3^. Threshold-based image segmentation was performed to obtain a 3D reconstruction and quantification of the ossification. µCT scans were visualized and rendered on Slicer 3D. Quantification of the heterotopic bone volume of in the injured muscles was obtained with the same software.

### Immunofluorescence

For immunofluorescence serial 10 µm thick muscle cryosections were thawed and fixed with 4% PFA in PBS for 10 min. Slides were then washed with PBS and permeabilized with 0.2% Triton and 1% BSA solution in PBS for 30 min, at RT. This was followed by blocking in 10% Donkey serum (DS) and 1% BSA solution in PBS, for 30 min, and incubation with primary antibodies (see Table 1) in 1% DS in PBS overnight, in a wet chamber, at 4 °C. The following day, slides were washed in PBS and incubated with secondary antibodies (Table 2) in 1% DS in PBS for 1 h at RT. Then, they were washed again, counterstained and mounted (Fluoroshield™ with 4’,6-diamidino-2-phenyl-indole (DAPI), Sigma-Aldrich, F6057). Slides were examined and images were recorded under a confocal microscope Zeiss LSM 710 equipped with an EC Plan-Neofluar 40x/1.30 Oil DIC M27. A minimum of 3 animals per group was used and at least 3 different fields per animal acquired. Counts from all fields corresponding to one animal were averaged. For quantification of cell numbers per field, individual cells were manually counted using the appropriate channels in combination with DAPI to identify nuclei using Fiji/ImageJ software.

**Table 1:** List of primary antibodies

Antibody (n. cat.)	Species	Dilution	Company
Mouse IgG Anti-F4/80 (MCA497)	Rat	1:50	BioRad
Mouse IgG Anti-Spp1 (PA5-141,129)	Rabbit	1:100	Invitrogen
Mouse IgG Anti-Sox9 (AB5535)	Rabbit	1:300	Millipore
Mouse IgG Anti-Sp7/Osterix (EPR21034)	Rabbit	1:300	Abcam
Mouse IgG Anti-iNOS (AB15323)	Rabbit	1:50	Abcam
Mouse IgG Anti-CD206 (AF2535)	Goat	1:100	R&D Systems
Human IgG Anti-Spp1 (AF1433)	Goat	1:25	R&D Systems
Mouse IgG Anti-MF20	Mouse	1:3	Developmental studies hybridoma bank

**Table 2:** List of secondary antibodies

Antibody	Dilution	Company
Alexa Fluor 488 IgG Donkey anti Rabbit (cat. A32790)	1:500	Invitrogen
Alexa Fluor 488 IgG Goat anti Mouse (cat. A-11001)	1:500	Life Technologies
Alexa Fluor 555 IgG Donkey anti Rabbit (cat. A32794)	1:500	Invitrogen
Alexa Fluor 555 IgG Donkey anti Goat (A32816)	1:500	Invitrogen
Alexa Fluor 647 IgG Donkey anti Rat (cat. A48272)	1:500	Invitrogen

### Cell isolation from skeletal muscle

Muscles were harvested and minced on a petri dish with a pair of scissors, incubated on a shaker at 37 °C adding 2 ml of enzyme mix (Calcium/Magnesium-Free PBS mixed with collagenase 1:15 (Sigma-Aldrich, C0130) and dispase 1:10 (Gibco, 17,105–041)) for 30 min. After the first incubation, a part of the supernatant was removed, 2 ml of enzyme mix added and the samples were left incubating on a shaker at 37 °C for 30 min. After the incubation, the suspension was homogenized using a syringe with a 16G needle and then with a 26G needle, until uniform without floating pieces of muscles. The suspension was then filtered through 40 µm cell strainers and 3 ml of CMF (Calcium/Magnesium-Free PBS, 10% FBS (Gibco, 10,270–106), 5% Pen/Strep (Gibco, 15,140–122), 2 mM EDTA) were added to block the enzymes. After centrifugation at 250 g for 10 min at rt, the supernatant was removed and the pellet was resuspended in 200 µl of red cell lysis buffer and left incubating for 10 min at rt. After the incubation, the red cell lysis buffer was blocked adding 1 ml of CMF.

### Single-cell RNA sequencing

For single-cell RNA sequencing, FOP and Acvr1^R206Hlox^;R26Sor^CreERT2^ mice (controls) were used respectively as FOP and control mice. We used three mice for each genotype to have three biological replicates. The sequencing was performed both at 5 and 7 days after the injury, for a total of 4 experimental conditions (control 5 days post injury, control 7 days post injury, FOP 5 days post injury and FOP 7 days post injury).

Gastrocnemii were dissected and digested as described above. To remove cell debris, the samples were processed with the Debris Removal Solution (Miltenyi, 130–109–398). Cell suspension was centrifuged at 300 g for 10 min at 4 °C, then supernatant was aspirated and the pellet was resuspended in 4 ml of cold Calcium/Magnesium-Free PBS and transferred in a 15 ml tube. Cold Debris Removal Solution (900 µl) was added and mixed by pipetting 10 times, then overlayed with 4 ml of cold Calcium/Magnesium-Free PBS without mixing the two phases. Cells were then centrifuged at 3000 g for 10 min at 4 °C. Supernatant was slowly aspirated and discarded and 15 ml of Calcium/Magnesium-Free PBS were added, followed by centrifugation at 1000 g for 10 min at 4 °C. Finally, supernatant was aspirated completely, and cells were resuspended in cold Calcium/Magnesium-Free PBS.

We counted the cells and confirmed that cell viability was higher than 90%. After obtaining a clean single-cell suspension, samples were processed on the Chromium platform (10X) using the Chromium Single Cell 3′ Library & Gel Bead Kit v3 kit (10X). After quality controls and quantification on TapeStation instrument (Agilent), libraries were sequenced on NextSeq500 platforms (Illumina) generating around 100000 reads/cell. Raw sequencing data was demultiplexed with the mkfastq application (Cell Ranger v.3.0.2). Reads were aligned to the reference genome and assigned to genes with cellranger count (Cell Ranger v.3.0.2, genome: refdata-gex-mm10-2020-A), with the expect-cells option set to 100000. The produced filtered matrixes were used for further bioinformatic analysis on R.

### Sample integration and cluster generation

Seurat v4.4 was used to analyse the 12 samples of this experiment and harmony v.1.2 was used to perform the integration of single cell genomics datasets, starting from a merged object scaled and normalized according to the default Seurat pipeline. PCA analysis and clustering were performed on the integrated dataset using the top 2000 highly variable genes and the first 30 PCs. The clustering was performed with the standard Seurat-v4 graph-based clustering approach [20], based on the functions FindNeighbours and FindCluster, where we set a resolution of 0.2. Cluster visualization was performed with UMAP dimensional reduction technique implemented in Seurat, which is based on uwot R package.

### Differential gene expression analysis

To investigate the identity of the obtained clusters we assessed the cluster’s marker genes, computed via the FindMarkers function with default parameters. We also used the data set proposed by McKellar et al. [21], using the TransferData Seurat function, which projects the PCA structure of a reference onto a query object, returning a predicted cell type annotation (see Fig. S1). To estimate the significance of the difference between the proportion of cells in the different clusters between FOP and Ctr samples we used scProportionTest (https://github.com/rpolicastro/scProportionTest).

After cell type identification, the differential gene expression (DGE) between FOP and Ctrl mice in FAPs and MPs cell type was performed with the FindMarkers function in Seurat, using default values (Wilcox test, logfc.threshold = 0.25 and min.pct = 0.1).

We then performed enrichment analysis of the differentially expressed genes with the CRAN enrichR package (v.2.1), which provides an R interface to the enrichR databases and statistics. We considered only genes with a log fold change > 0.25 and < − 0.25 and adjusted P value < 0.05.

### Trajectory analysis

We used the Monocle2 R package (v2.18) to perform a trajectory analysis of MPs (MPs) subpopulations post-injury [22]. First, we subset the cells labelled as MPs from the Seurat dataset and across all time points and samples. Second, we performed the standard Seurat-v4 graph-based clustering approach [20] on this subset to identify new subpopulations in the data. We then used the Seurat FindAllMarkers function to find differentially expressed genes that characterize the subpopulations. We selected the most expressed genes based on fold-change expression with a minimum of log2(0.8) and adjusted p value of 0.01.

Unsupervised ordering was obtained with Monocle2 DDRTree algorithm based on variable genes detected previously using Seurat’s function FindVariableGenes with default parameters. The root of the trajectories was computationally determined using the GM_state function in Monocle2 to identify the State with highest number of Precursor (monocyte-like) which had been defined as starting point of the differentiation process by RNA velocity. Plot_cell_trajectory function was used to plot the minimum spanning tree on cells and highlight pseudotime and subpopulation distribution.

### Interference and analysis of cell–cell communication

To identify conserved and altered communication networks in control and FOP mice, intercellular communication networks were modelled based on the abundance of known ligand-receptor transcript pairs with CellChat (version 1) [23].

### Bone marrow derived MP (BMDMs) isolation and polarization

BMDMs were isolated from femur and tibia of FOP and control mice as in [24]. To polarize BMDMs, they were seeded in growth medium (DMEM High glucose (Sigma-Aldrich, D5796), 10% FBS (Gibco, 10,270–106), 1% Pen/Strep (Gibco, 15,140–122)). After cells have attached, the medium was changed, cells were gently washed with Calcium/Magnesium-Free PBS and growth medium supplemented with polarizing factors was added: IFNγ 50 ng/ml (R&D systems, 485-MI-100) for pro-inflammatory MPs and dexamethasone 100 nM (Sigma Aldrich, D4902) for anti-inflammatory MPs.

### Fibro-adipogenic progenitor (FAP) isolation

FAPs were isolated from the hindlimbs muscles as in [24]. Gastrocnemius, tibialis anterior and quadriceps were digested as previously described. Upon reaching a homogeneous cell suspension, FAPs were magnetically sorted using MACS columns (Miltenyi LD separation columns, 130–042–901; Miltenyi MS Separation columns, 130–042–201) and magnetic beads in three steps: first we selected the CD45^−^ population (Miltenyi, 130–052–301), then Sca1^+^ cells (Miltenyi, 130–106–641) and finally the PDGFRα^+^ (Miltenyi, 130–101–502) population in order to sort FAPs as CD45^−^/Sca1^+^/PDGFRα^+^ cells. FAPs were then cultured in DMEM High Glucose (Sigma-Aldrich, D5796) supplemented with 20% FBS (Gibco, 10,270–106), L-glutamine and 1% Pen/Strep (Gibco, 15,140–122).

### FAP osteogenic differentiation

FAPs derived from controls and FOP mice were cultured in osteogenic medium: DMEM High Glucose (Sigma-Aldrich, D5796), 2.5% FBS (Gibco, 10,270–106), 1% Pen/Strep (Gibco, 15,140–122), 50 µg/ml ascorbic acid (Fujifilm Wako Chemicals, 013–19641), 100 nM dexamethasone (Sigma Aldrich, D4902), 10 mM β-glycerophosphate (Sigma-Aldrich, G9422). The treatment was carried on for 6 days, changing the medium once every 3 days. Osteogenic differentiation was then evaluated by Alizarin red staining. The chamber slides were fixed with 4% paraformaldehyde for 10 min, then washed with Calcium/Magnesium-Free PBS and stained with Alizarin red solution (Sigma Merck, 2,003,999). In parallel, Alkaline phosphatase (ALP) activity was assessed using a histochemical staining kit (86R-1KT, Sigma Aldrich). Images were acquired on Olympus BX63 microscope (Magnification 6.3X, N.A. 0.4) and on Zeiss CellObserver® microscope (Magnification 10X, N.A. 0.3).

### FAP treatment with conditioned medium (CM) derived from MPs

To prepare the CM, both control and FOP MPs were polarized 3 days as above. Polarizing medium was then removed, cells were washed with PBS and starvation medium was added to obtain MPs CM, composed as follows: DMEM High Glucose (Sigma-Aldrich, D5796), 2.5% FBS (Gibco, 10,270–106), 1% Pen/Strep (Gibco, 15,140–122). On the same day, FAPs were seeded in growth medium and let adhere overnight.

After 24 h, the CM was ready to be collected in 15 ml tubes and centrifuged at 450 g to remove debris and dead cells. We added the osteogenic differentiating factors to the CM (50 µg/ml ascorbic acid (Fujifilm Wako Chemicals, 013–19641), 100 nM dexamethasone (Sigma Aldrich, D4902), 10 mM β-glycerophosphate). Growth medium was then removed from FAPs and cells were washed with PBS. CM was added to FAPs and was renewed every 3 days. After 6 days, FAPs were used for different applications. 

### RNA extraction and rt-qPCR

6 × 10^5^ FAPs or 10^6^ BMDMs were plated in 6 well plates. After treatment, the medium was removed and 350 µl of RLT buffer + β-mercaptoethanol 1:100 from RNeasy Plus Mini Kit (Qiagen, 74,134) was added to FAPs directly into the wells. Cells were then detached using a cell scraper. Lysates were directly loaded into a QIAshredder spin column (Qiagen, 79,656) and centrifuged for 2 min at the maximum speed. Total RNA was isolated using RNeasy Plus Mini Kit, following manufacturer instructions.

Total RNA was quantified by measuring the absorbance at 260 nm in a Nanodrop (Thermo Fisher Scientific) and RNA purity was evaluated by the ratio of absorbance at 260 and 280 nm. RNA samples were reverse transcribed to cDNA with the LunaScript RT SuperMix Kit (New England Biolabs, E3010L), using the Thermocycler 2027 (Applied Biosystems). Real-time rt-qPCR was carried out using the Luna Universal qPCR Master Mix (M3003X, New England Biolabs) in a QuantStudio™ 7 Flex Real-Time PCR System (Applied Biosystems). Amplification reactions were performed in duplicate. To check specificity, a dissociation curve was derived at the end of each run. Controls lacking reverse transcriptase were included to ensure no genomic DNA contamination during preparations. Primers used are listed in Table 3. Quantification was performed using the relative 2^−ΔΔCT^ method and 28S expression was used as internal control.

**Table 3:** List of primers for rT q-PCR

Gene	Sequence
SOX9	FW: 5’ – TCCAGCAAGAACAAGCCACA – 3’
	RV: 5’ – CGAAGGGTCTCTTCTCGCTC – 3’
OSX	FW: 5’ – ACCAGAAGCGACCACTTGAG – 3’
	RV: 5’ – TTGGCTTCTTCTTCCCCGAC – 3’
NOS2	FW: 5’ – AGCCAAGCCCTCACCTACTT – 3’
	RV: 5’ – TCTCTGCCTATCCGTCTCGT – 3’
CD206	FW: 5’ – TTGCACTTTGAGGGAAGCGA – 3’
	RV: 5’ – AGTCCAATCCAGAGTCCCGA – 3’
INHBA	FW: 5’ – GGCTCTTCCTGAAAGTCCCC – 3’
	RV: 5’ – GCAAATGTTGACCTTGCCGT – 3’
SPP1	FW: 5’ – GATCAGGACAACAACGGAAAGG – 3’
	RV: 5’ – GCTGGCTTTGGAACTTGCTT – 3’
28S	FW: 5’ – AAACTCTGGTGGAGGTCCGT – 3’
	RV: 5’ – CTTACCAAAAGTGGCCCACTA – 3’

### Enzyme-Linked Immunosorbent Assay (ELISA)

ELISA was performed to detect SPP1 (Abcam, ab100734) and Activin A (Abcam, ab119569) in cell medium derived from MPs, according to experimental needs. The assays were performed following the protocols provided by the manufacturer. Supernatants were diluted as following: to detect Spp1 concentration, MPs’ cell media were diluted 1:400. To detect Activin A, media were diluted 1:10. O.D. absorbance was read at 450 nm with a FluoStar OMEGA plate reader (BMG Labtech).

### Western Blot

FAPs were lysed in RIPA buffer with protease inhibitor cocktail (1:500, Sigma Aldrich, P8849) and phosphatase inhibitor cocktail (1:100, Cell Signaling, 5870). Protein concentration was determined by Bradford Assay (BioRad, 5,000,205). Cell lysates were separated on Precast Tris–Glycine gels (4–20%, Clinisciences, DG101-420-10w-02) by SDS-PAGE electrophoresis and transferred to Nitrocellulose blotting membrane (Amersham). Primary antibodies against SMAD 1/5 (Invitrogen, PA5-80,036), pSMAD 1/5 (Cell Signaling, 9516) and vinculin (Cell Signaling, 4650) were used at 1:1000 dilution. Membranes were then incubated with goat-anti rabbit horseradish peroxidase-conjugated secondary antibody (Invitrogen). Proteins were detected by chemiluminescence using Clarity Max Western (BioRad) and visualized with CHEMIDOC® Imaging System (BioRad).

### FAP treatment with SPP1

Control and FOP FAPs were seeded and let adhering o/n. FAPs seeding density was determined depending on the experiment as previously described. The day after, cells were treated with SPP1 200 ng/ml (R&D Systems, 441-OP-050) diluted in low serum medium. The medium was changed once every 3 days. After 6 days of treatment, FAPs were either stained for Alizarin red or ALP staining, or collected for RNA extraction and rt-qPCR analysis or Western blot analysis as previously described.

### *In vitro* inhibition of SPP1

Control and FOP FAPs were cultured as above with CM derived from FOP or Control anti-inflammatory MPs in presence with either 2.5 μg/mL anti-SPP1 neutralizing mAb (R&D Systems, AF1433), or the same concentration of an isotype-matched mouse IgG as control (R&D Systems, AB-108-C). The medium was renewed once every 3 days. After 6 days of treatment, FAPs were either stained for Alizarin red or ALP staining, or collected for RNA extraction and rt-qPCR analysis as previously described.

SPP1 inhibition was also evaluated in vitro in FAP using of the OPN expression inhibitor 1 (OPN-IN-1) (HY-146064, MedChemExpress). FOP FAPs were cultured as above with FOP CM in presence of either 25 μg/mL OPN-IN-1 or DMSO (control). After 6 days of treatment, FAPs were stained for ALP.

### *In vivo* inhibition of SPP1

To achieve inhibition of SPP1 *in vivo*, HO was induced in 8-week-old FOP mice by pinch injury of the gastrocnemius muscle, as described above. Mice were then randomly assigned to two groups: one group received 5 mg/kg of the OPN expression inhibitor 1 (OPN-IN-1; HY-146064, MedChemExpress) by intraperitoneal injection on the day of muscle injury and again at 3, 6, and 9 days post-injury, while control mice received an equivalent volume of vehicle, as described in[25, 26]. μCT analyses were performed at days 14 and 21 post-injury to monitor HO formation. Animals were euthanized 21 days after pinch injury, and gastrocnemius muscles were collected for histological analyses.

### Statistical analysis

Differences between the two experimental groups were compared by Student’s t-tests using GraphPad Prism 8. Statistical significance was accepted for comparisons where P < 0.05. Values are presented as means ± SD.

## Results

### Ectopic bone development in Acvr1R206H^flox/flox^;Gt(ROSA26)Sor^CreERT2^ mice upon muscle injury is influenced by MP infiltration

Acvr1^R206H^;Gt(ROSA26)^SorCreERT2^ mice (hereafter referred as FOP mice), in which recombination of the Acvr1^R206H^ allele has been driven by the ubiquitously expressed and tamoxifen dependent Gt(ROSA26)^SorCreERT2^ promoter, reproduce the pathological features of FOP. This can be achieved by triggering localized inflammatory response in mice by mechanical pinching the gastrocnemius muscle, that induces a reproducible injury, thus recapitulating the acute flare-ups observed in FOP patients. Quantitative µCT analysis showed the progressive development of ectopic bone formation, with bone volume increasing by approximately 50% between days 14 and 21 (34.5 ± 6.4 mm^3^ vs 54.4 ± 4.9 mm^3^, 14 days vs 21 days, P < 0.05) (Supplementary Fig. S1A). No HO was observed in Acvr1^R206Hlox^ Gt(ROSA26)^SorCreERT2^ mice that did not receive tamoxifen (control mice, Ctr) (Supplementary Fig.S1A).

Previous studies have suggested a contribution of MPs and mast cells in HO development in a FOP mouse model [27] To further investigate the role of infiltrating MPs during lesion formation in our experimental setting, we performed circulating monocyte/MP depletion using injection of clodronate liposomes, which induce apoptosis in phagocytic cells upon internalization [26]. Four intravenous injections of clodronate loaded liposomes were administered—starting one day before injury and every other day thereafter. A group of mice received PBS-containing liposomes (Sham), and both groups were monitored for 21 days. FACS analysis of peripheral blood showed consistent reduction of CD45^+^/CD11b^+^ (25.5 ± 3.3% vs 15.6 ± 2.6%, Sham vs CLL, P < 0.05) (Supplementary Fig. S1B) in line with our previous studies [13, 28, 29].

**Fig. 1:**
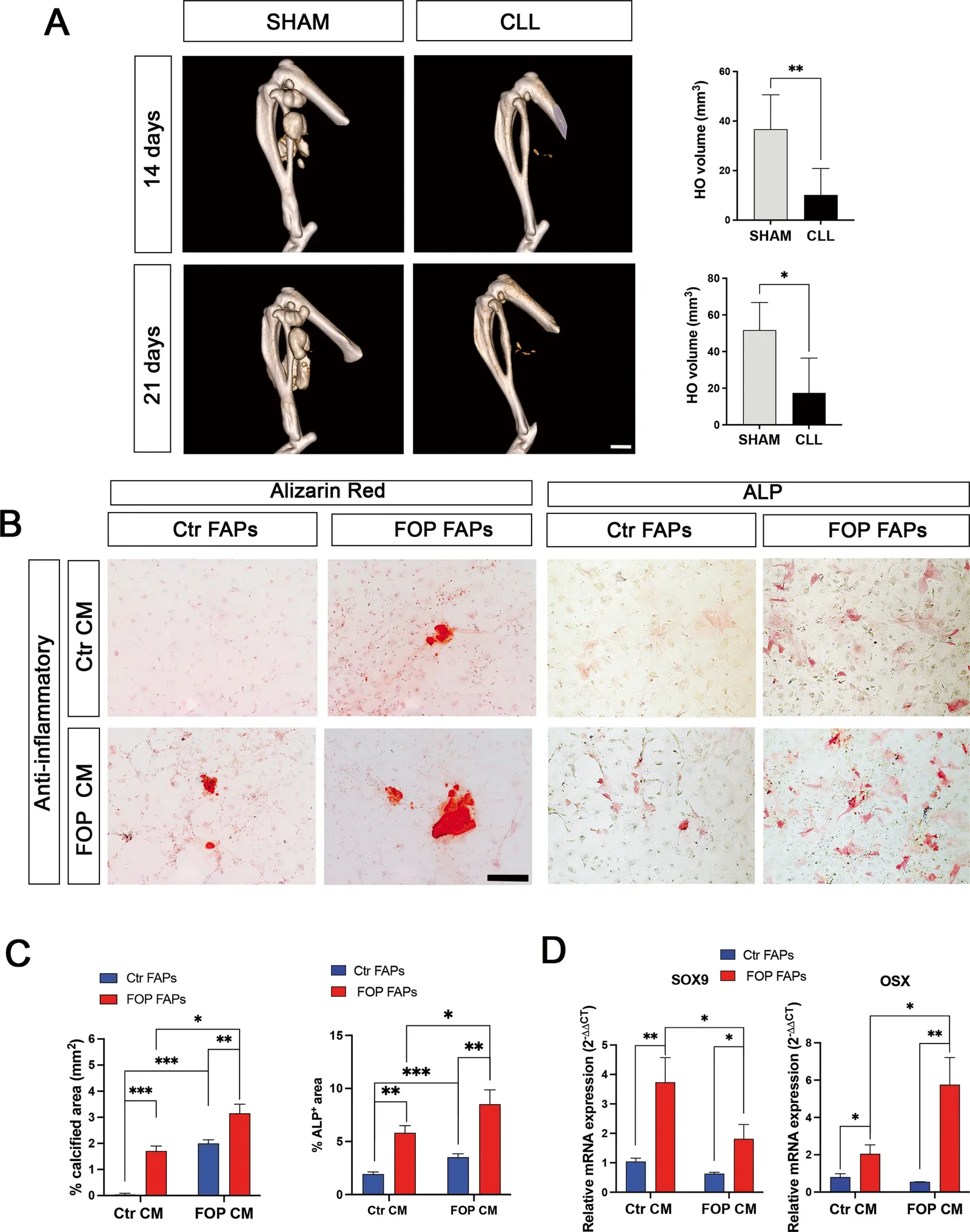
“Impact of MPs during injury response in Ctr and FOP mice *in vivo* and indirect co-culture with FAPs” A) Micro-CT of injured FOP mice treated with control (SHAM) or clodronate (CLL) liposomes at 14 and 21 days post injury. Data are presented as mean ± SD (n = 3). Statistically significant differences are indicated as * and **, representing P < 0.05 and P < 0.01 respectively (two-tailed unpaired Student’s t-test). Scale bar: 5 mm. B) Ctr and FOP FAPs treated for 6 days with conditioned medium derived from Ctr and FOP anti-inflammatory MPs and stained for Alizarin Red (calcified areas) (Magnification 6.3X, Scale Bar: 200 μm) or Alkaline Phosphatase (Magnification 10X, Scale Bar: 100 μm). C) Quantification of area positive for Alizarin Red staining or for ALP staining. Data are presented as mean ± SD (n = 3). Statistically significant differences are indicated as *, **, *** and **** representing P < 0.05, P < 0.01, P < 0.001 and P < 0.0001, respectively. (two-tailed paired and unpaired Student’s t-tests). D) Gene expression levels of chondro/osteogenic markers *Sox9* and *Osx* in Ctr and FOP FAPs treated with MPs conditioned medium. Results are normalized to 28S gene used as internal control. Data are presented as mean ± SD (n = 3). Statistically significant differences are indicated as * and ** representing P < 0.05, and P < 0.01 respectively (two-tailed paired and unpaired Student’s t-tests)

µCT analysis revealed a significant reduction in ectopic bone volume in clodronate-treated mice at both 14- (36.7 ± 13.9 mm^3^ vs 10.1 ± 10.6 mm^3^, Sham vs CLL, P < 0.01) and 21-days post-injury (51.7 ± 15 mm^3^ vs 17.4 ± 18.9 mm^3^, Sham vs CLL, P < 0.05) (Fig. 1A). These results indicate that MP depletion is sufficient to delay the endochondral ossification process and markedly reduces ectopic bone formation, highlighting the pivotal contribution of MPs to HO progression in FOP mice.

Given that FAPs are the principal cell type responsible for initiating HO in FOP [30], we next investigated the MP–FAP interaction during osteogenesis in vitro. FAPs and BMDMs were isolated from FOP and control mice. FAPs were then cultured with conditioned media (CM) derived from BMDMs of either genotype, polarized in vitro toward pro- or anti-inflammatory phenotypes.

FOP FAPs exposed to conditioned media (CM) from BMDMs exhibited accelerated osteogenic differentiation with respect to control FAPs (Fig. 1B–C; Fig. S1C). Notably osteogenic differentiation was enhanced if the CM was derived by FOP BMDMs (Fig. 1B–C; Fig. S1C). This effect was evident through higher Alizarin Red staining (1.7 ± 0.2% vs 3.1 ± 0.3%, CM Ctr/FAPs FOP vs CM FOP/FAPs FOP, P < 0.05) (indicating calcium deposition, Fig. 1B), Alkaline Phosphatase staining (5.8 ± 0.7% vs 8.5 ± 1.3%, CM Ctr/FAPs FOP vs CM FOP/FAPs FOP, P < 0.05) (Fig. 1B-C), and increased expression of osteogenic transcription factor *Osx* (2 ± 0.4 vs 5.7 ± 1.4, CM Ctr/FAPs FOP vs CM FOP/FAPs FOP, P < 0.05) (Fig. 1D). Consistently the expression of the chondrogenic transcription factor *Sox9* is decreased in these FOP FAPs treated with FOP CM respect to those treated with control CM (Fig. 1D) (3.7 ± 0.8 vs 1.8 ± 0.4, CM Ctr/FAPs FOP vs CM FOP/FAPs FOP, P < 0.05).

Together, these findings suggest that soluble FOP MP-derived factors promote the ectopic bone formation characteristic of FOP.

### Single-cell RNA sequencing reveals altered cell clusters in response to muscle injury in FOP mice

To investigate the early molecular and cellular events leading to HO in FOP mice, we performed single-cell RNA sequencing (scRNA-seq) on gastrocnemius muscles from control and FOP mice at 5 and 7 days post-pinch injury. Based on previous studies in FOP and skeletal muscle regeneration, these time-points were selected as representative early stages of lesion development, characterized by active inflammatory remodeling, MP polarization, stromal cell expansion, and initiation of osteochondrogenic programs prior to overt ectopic mineralization [13, 26, 27, 29].

**Fig. 2:**
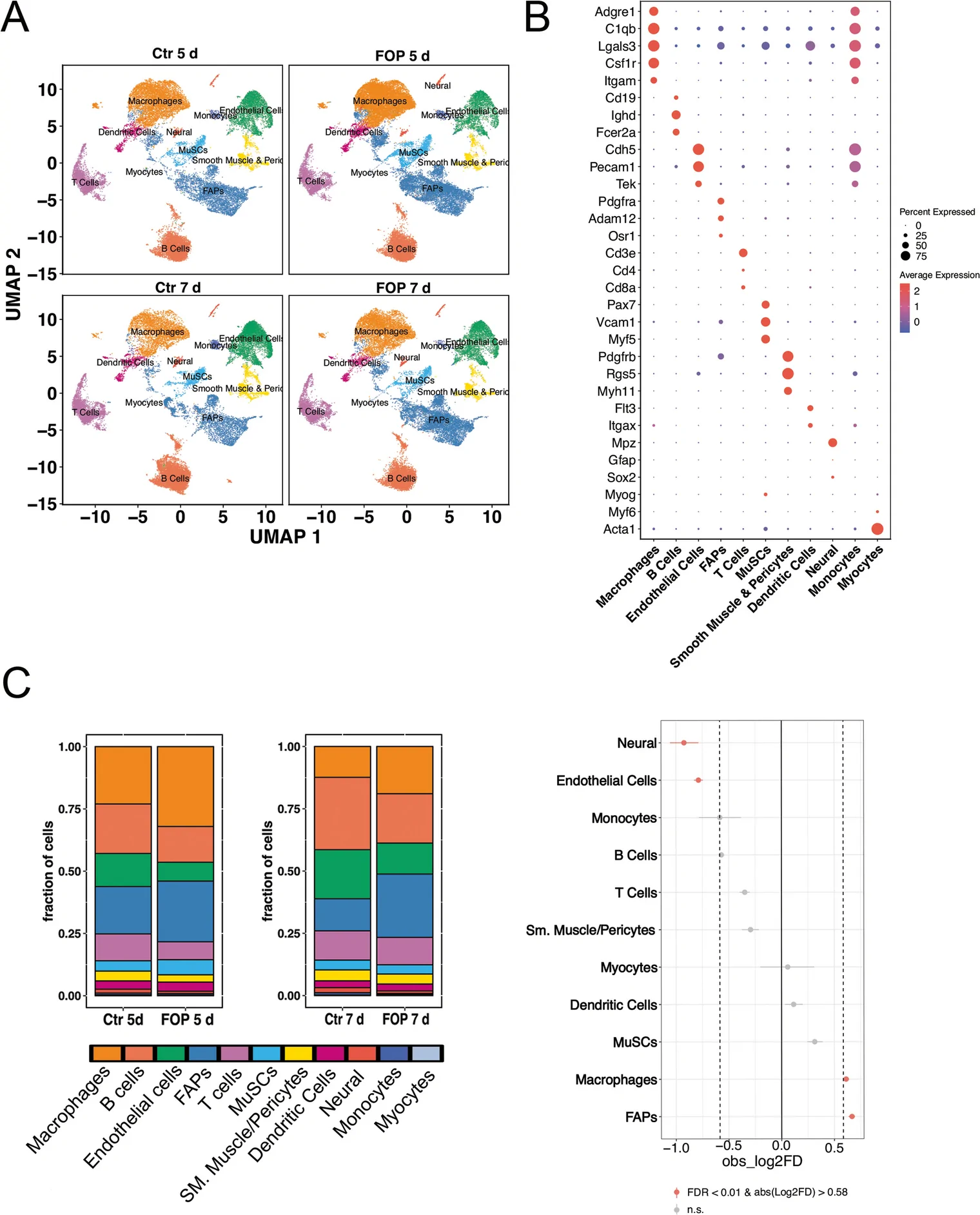
“Single-cell RNA sequencing shows an increase in MPs and FAPs in FOP mice in response to injury”. A) UMAP embedding of all sequenced samples divided by genotyping and timepoint, coloured by individual cluster identities and annotated according to markers’ gene expression. B) DotPlot demonstrating cell-type marker gene expression used for meta-cluster classification. Dot size indicates the percentage of cells expressing each gene, and dot colour represents the average expression levels. C) Relative proportion and frequency of cell types per condition (left panel) and significance of cluster abundance differences in FOP *versus* Ctr samples (right panel)

Cell clusters were identified using a shared nearest neighbour (SNN) modularity optimization clustering algorithm, which groups cells based on differences in their principal component (PC) values and a resolution parameter. Unsupervised clustering defined 30 principal components, which were then labelled and merged into 11 main clusters based on canonical skeletal muscle gene expression profiles (Fig. 2A, Supplementary Fig.S2A,) [21]. Marker genes and their relative expression for each cluster are presented in the dot plot and heatmap in Fig. 2B and Supplementary Fig. S2B respectively (and Supplementary Table S1).

MPs formed the largest immune cell cluster, marked by high expression of *Apoe, Aif1*, *Adgre1* (F4/80), and *Csf1r*, also expressed in monocytes together with *CD68*. B cells expressed *Cd19* and *Cd79a* while T cells expressed *Cd3* and *Tcrb2*. Endothelial cells were identified by *Cdh5* (VE-cadherin) and *Pecam1* (CD31) expression and FAPs by *Pdgfra*. Muscle stem cells (MuSCs) and myoblasts were characterized by *Pax7* and *Myf5*, whereas mature myocytes expressed *Acta1*. Smooth muscle cells and pericytes expressed *Rgs5* and *Pdgfrb*. Dendritic cells expressed *Cd209a* and *Cd74*, and a neural/glial cluster was identified by *Sema3b*, *Mpz* and *Mbp* expression. The cell populations identified were consistent with prior scRNA-seq studies of regenerating skeletal muscle [21, 31, 32].

Comparative analysis between FOP and control mice revealed substantial shifts in cellular composition across conditions and timepoints (Fig. 2C). FOP mice showed a statistically significant increase in both FAPs and MPs at 5- and 7-days post-injury, underscoring their potential contribution to HO development (Fig. 2C). Conversely, a significant decrease in endothelial cells was observed in FOP samples. Given their known role in promoting MuSC-mediated muscle regeneration after injury [14], this reduction suggests impaired muscle repair in FOP. These findings support a model in which early injury responses in FOP are dominated by a sustained inflammation and a profibrotic cellular environment that may drive ectopic bone formation.

Based on these observations, we focused our downstream differential gene expression (DGE) and functional analyses on FAPs and MPs, given their expansion and potential crosstalk in the evolving ossification niche (Fig. 1B–C; Supplementary Fig. S1C).

### FAPs actively contribute to bone formation by upregulating ossification genes in FOP mice

**Fig. 3:**
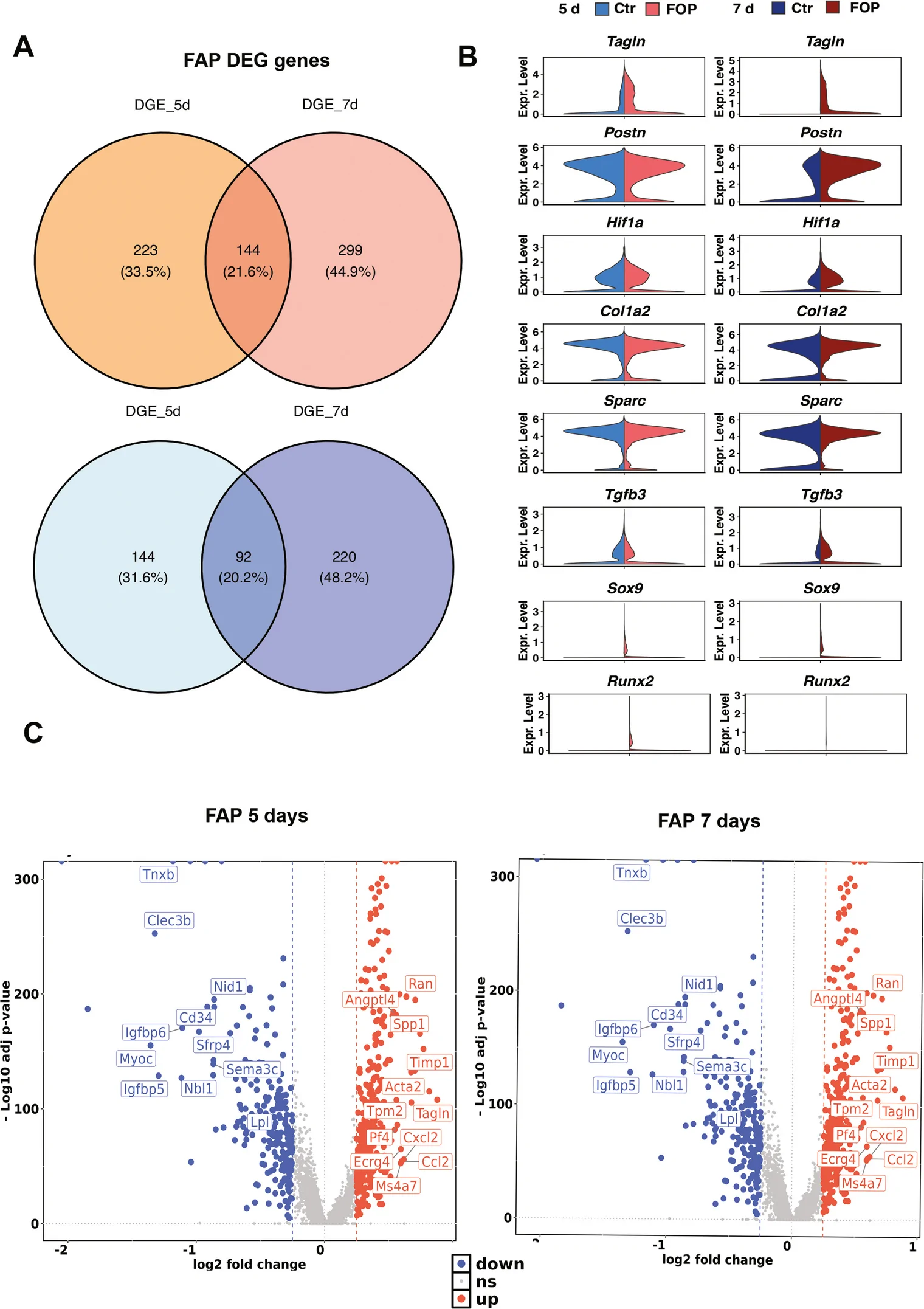
“Transcriptomic characterization of Ctr and FOP FAPs”. A) Venn diagrams representing the intersection of significantly upregulated (left panel) and downregulated (right panel) genes in the comparison FOP *versus* Ctr at 5 and 7 days. B) Violin Plots showing normalized and log-transformed expression of FAPs specific upregulated genes, in the comparison FOP *versus* Ctr at 5 and 7 days. C) Volcano Plots showing statistical significance (-log10[P value]) *versus* fold change (log2[fold change]) of RNA-seq data from FOP *versus* Ctr FAPs at 5 days (left panel) and 7 days (right panel). Genes with increased expression are shown in red, and genes with decreased expression are shown in blue. The selection of up-regulated and down-regulated genes were based on fold-change expression with a minimum of log2[0.25] and adjusted p-value of 0.05

The role of FAPs in HO is well established [18, 30, 33]. To investigate the molecular programs underlying the increased abundance and osteogenic shift of FAPs in FOP, we performed differential gene expression (DGE) analysis followed by Enrichment Analysis using the GO and KEGG databases via the CRAN enrichR package (v.2.1). In FOP FAPs, we identified 223 and 299 upregulated genes as compared to controls at 5- and 7-days post-injury, respectively (p < 0.05), while fewer genes were downregulated (144 and 220, respectively) (Fig. 3A, Supplementary Table S2, S3).

At 5 days post-injury, FOP FAPs exhibited significant enrichment in inflammatory pathways, including responses to IL-1, IL-17, and IFN-γ, as well as NF-κB signaling, with increased expression of pro-inflammatory chemokines such as CCL2 and CXCL2 (Fig. 3C, Supplementary Fig.S3A-B). In parallel, we observed upregulation of the HIF-1 signaling pathway at both 5 and 7 days, suggesting an enhanced hypoxic environment in FOP muscle that may facilitate chondrogenic and osteogenic commitment (Fig. 3B-C, Supplementary Fig.S3B, Supplementary Tables S2−3).

We also observed a downregulation of the *Lpl* gene (Fig. 3C), involved in FAP adipogenesis, further strengthening the hypothesis by which FAPs are committed towards chondro-osteogenic differentiation [34].

By day 7, FOP FAPs further upregulated genes associated with fibrosis, extracellular matrix (ECM) remodeling, and collagen organization—including *Acta2*, *Fn1*, and *Timp1* (Fig. 3B-C; Fig. S3A-B)—indicating a profibrotic response to muscle injury. Concomitantly, the expression of key osteochondral transcription factors, such as *Sox9* and *Runx2*, was elevated, pointing to ongoing endochondral ossification. Additional genes implicated in osteogenesis and ECM remodeling, including *Sparc*, *Postn*, *Tnc* and *Tagln*, were also upregulated (Fig. 3B-C; Supplementary Fig. S3A-B). Notably, *Tagln* (transgelin) and *Sparc* have previously been linked to FAP osteoblast and adipocyte differentiation downstream of TGF-β signaling [35–38].

Together, these data highlight a shift in FOP FAPs toward a profibrotic and osteogenic phenotype, with and increase sensitivity to inflammatory cues, making them more prone to respond to MP-derived signals.

### FOP MPs display an altered inflammatory profile after muscle injury

**Fig. 4:**
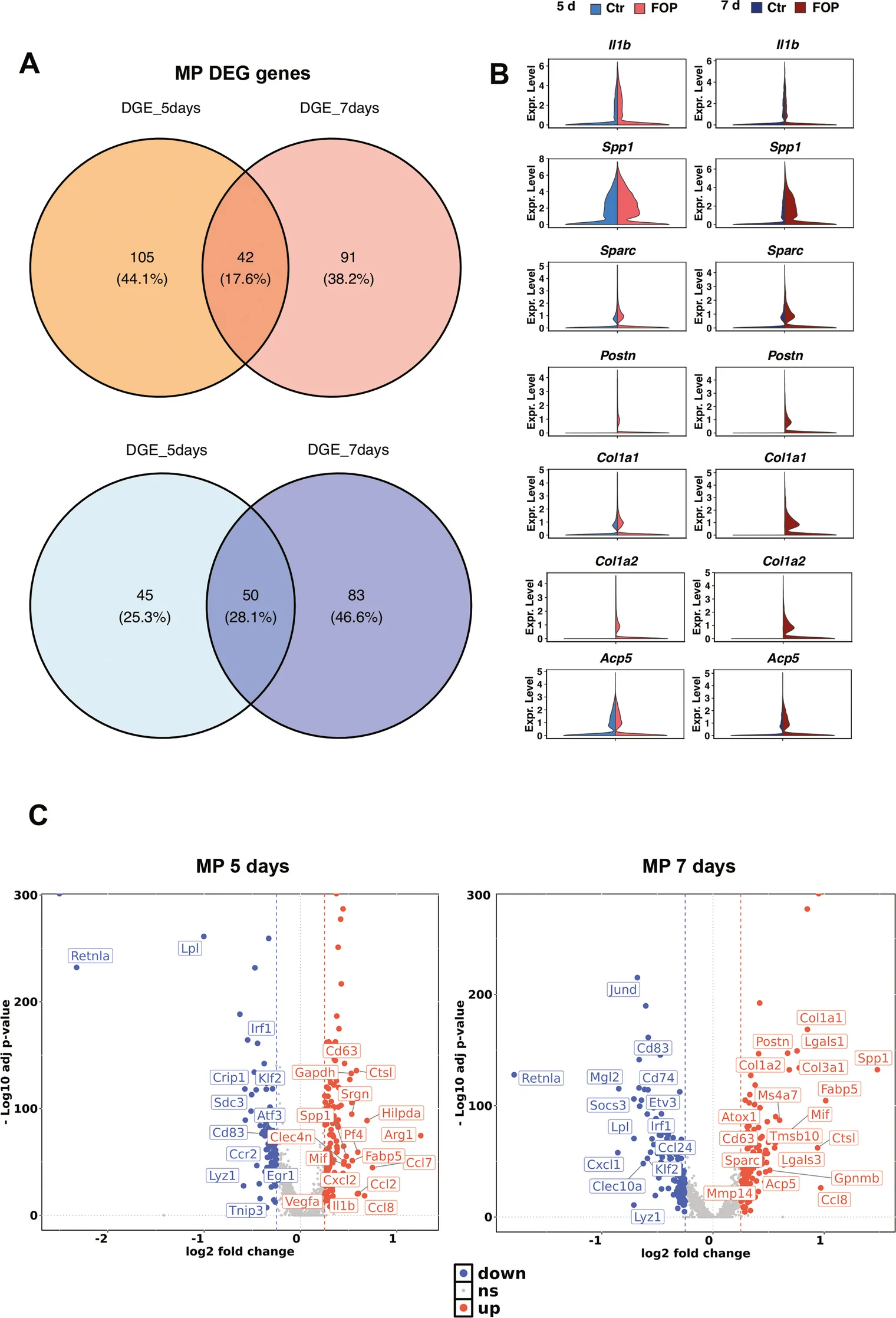
“Transcriptomic characterization of Ctr and FOP MPs”. A) Venn diagrams representing the intersection of significantly upregulated (left panel) and downregulated (right panel) genes in the comparison FOP *versus* Ctr at 5 and 7 days. B) Violin Plots showing normalized and log-transformed expression of MPs specific upregulated genes, in the comparison FOP *versus* Ctr at 5 and 7 days. C) Volcano Plots showing statistical significance (-log10[P value]) *versus* fold change (log2[fold change]) of RNA-seq data from FOP *versus* Ctr MPs at 5 days (left panel) and 7 days (right panel). Genes with increased expression are shown in red, and genes with decreased expression are shown in blue. The selection of up-regulated and down-regulated genes were based on fold-change expression with a minimum of log2[0.25] and adjusted p-value of 0.05

The role of MPs in HO has been only marginally explored in the context of FOP. To gain further insight, we performed differential gene expression (DGE) analysis on MP subsets, followed by Enrichment Analysis using the Gene Ontology (GO) and KEGG databases via the CRAN enrichR package (v.2.1). In FOP MPs, we identified 105 and 91 upregulated genes compared to controls at 5- and 7-days post-injury, respectively (p < 0.05), with a smaller number of downregulated genes at both timepoints (45 and 82, respectively) (Fig. 4A, Supplementary Tables S4−5).

At 5 days post-injury, GO enrichment analysis revealed the upregulation of genes involved in classical pro-inflammatory signaling pathways, such as IL1β, TNF-⍺, and NF-κB with significant overexpression of *Ccl7*, *Ccl8*, and *Il1β*. This inflammatory profile was accompanied by an increased expression of hypoxia-responsive genes, including *Hilpda* (Fig. 4B-C, Fig. S4A-B), consistent with previous evidence that the combination of hypoxia and inflammation promotes HO development [39].

Analysis of highly deregulated genes in FOP MPs at both timepoints revealed a consistent upregulation of *Spp1*, *Sparc*, and *Postn* (Fig. 4B-C, Supplementary Fig. S4A-B), which encode Osteopontin, Osteonectin, and Periostin, respectively—key extracellular matrix proteins involved in tissue remodeling and ossification [37]. Of note, *Postn* expression is regulated by mechanical stimuli in MPs via TGF-β activation, which also induces anti-inflammatory polarization [40], linking the physical microenvironment to MP phenotype and function. In addition, we observed upregulation of *Cd63* and *Gpnmb*, two genes commonly associated with fibrotic and tumor-associated MPs and known to contribute to tissue remodeling and pro-tumorigenic activity [41] (Fig. 4B-C) further highlighting the activated and tissue-modifying phenotype of FOP.

By 7 days post-injury, this activated profile became more pronounced, with significant upregulation of collagen-encoding genes *Col1a1*, *Col1a2*, and *Col3a1* (Fig. 4B-C, Supplementary Fig. S4A-B). These genes are central to the formation of a collagen-rich ECM that supports endochondral ossification, in line with prior studies showing that type I Collagen promotes the formation of a cartilaginous scaffold essential for bone formation [42].

Moreover, among the top upregulated genes at this later timepoint was *Acp5*, which encodes tartrate-resistant acid phosphatase (TRAP), a hallmark osteoclastic enzyme involved in bone resorption [43]. This finding was reinforced by the upregulation of several cathepsin genes (*Ctsl*, *Ctsd*, *Ctsz*) in FOP MPs, proteases that play key roles in degrading the organic matrix of bone and are typically expressed by active osteoclasts [44] (Fig. 4B-C, Supplementary Fig. S4A-B).

This first analysis indicated that FOP MPs acquired a highly plastic phenotype, simultaneously exhibiting inflammatory, osteoclast-like, and tissue-remodeling features that may contribute to the initiation and progression of heterotopic ossification.

**Fig. 5:**
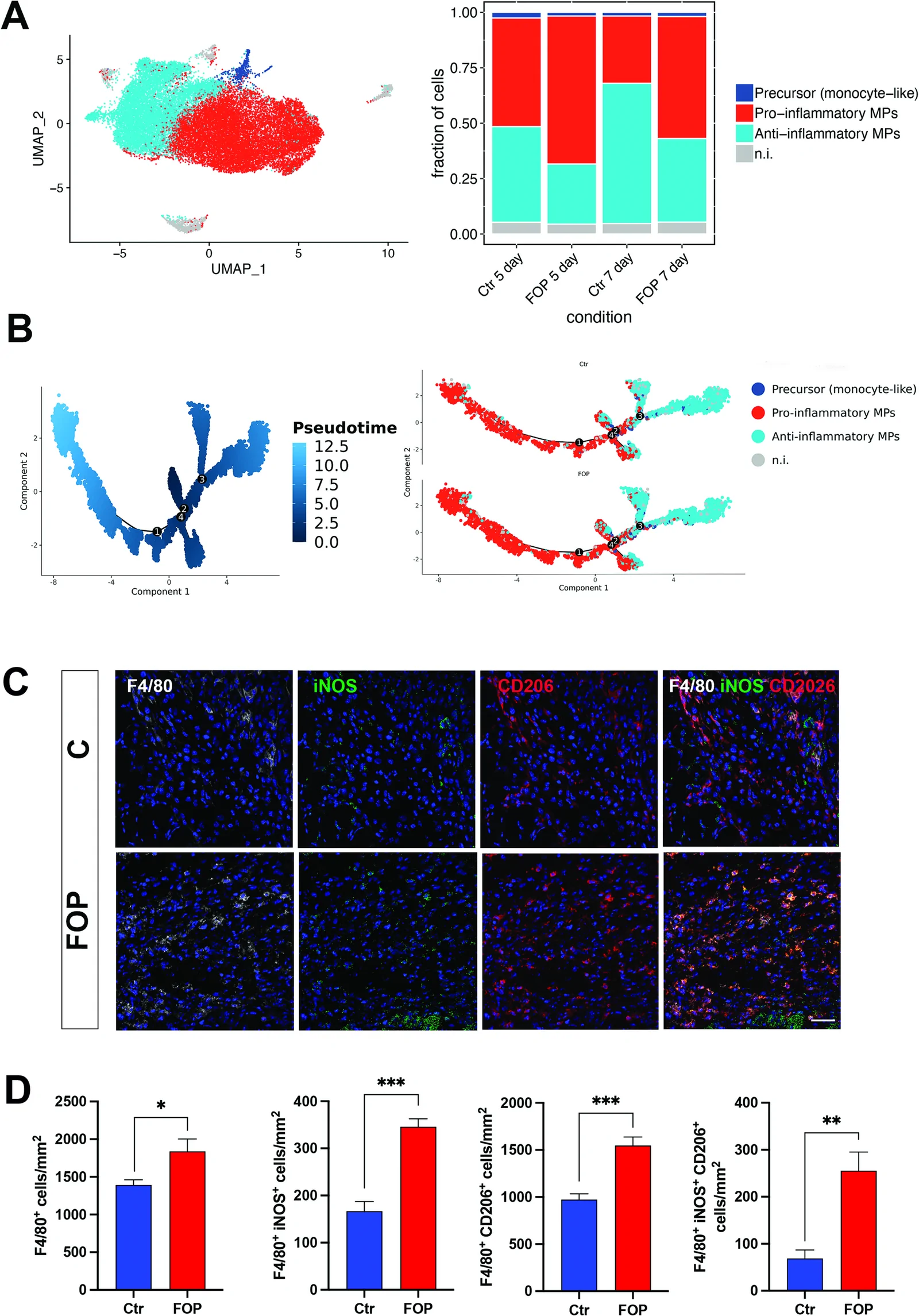
“Subclustering and trajectory analysis of monocytes and MPs”. A**)** Left—UMAP plot of subpopulations of MPs. Cells are colored according to their assigned subcellular identity, including precursor (monocyte-like) cells, pro-inflammatory MPs, anti-inflammatory MPs, and non-identified cells (n.i.). Right—Relative abundance of macrophage sub-populations in control (Ctr) and mutated (FOP) samples at 5 and 7 days post-injury. B) Monocle2 trajectory analysis of macrophage cells projected into reduced-dimensional space. Left panel: cells are colored by pseudotime, indicating the inferred progression from precursor/monocyte-like states toward differentiated MP phenotypes. Black points and connecting lines mark branch points and the main developmental trajectory. Right panels: the same trajectory is shown separately for Ctr and FOP samples, with cells colored by MP subtypes, highlighting differences in lineage progression and the balance between pro-inflammatory and anti-inflammatory MP states between the two conditions. C) Immunofluorescence staining of gastrocnemius muscle cross-sections of Ctr and FOP mice 7 days post muscle injury using specific antibodies for F4/80 (MPs, white), iNOS (M1-like MPs green) and CD206 (M2-like MPs, red). Nuclei were counterstained with DAPI (blue). Magnification 40X. Scale bar 40 μm. D) Quantification of F4/80^+^, F4/80^+^ iNOS^+^, F4/80^+^ CD206^+^ and F4/80^+^ iNOS^+^ and CD206.^+^ cells in gastrocnemius muscle cross-section of FOP and Ctr mice 7 days post muscle injury. Data are presented as mean ± SD (n = 3). Statistically significant differences are indicated as ∗, ∗ ∗, and ∗ ∗ ∗, representing P < 0.05, P < 0.01, and P < 0.001 respectively (two-tailed unpaired Student’s t-test)

To gain deeper insight into the alterations along the monocyte and MPs axis in FOP, we performed subclustering of monocyte and MPs using an optimal clustering strategy, coupled with supervised annotation based on a curated set of literature-derived marker genes [21]. This analysis revealed a notable enrichment of inflammatory monocytes in FOP mice, while anti-inflammatory MPs were more prevalent in controls. This difference, particularly pronounced at 7 days post-injury, suggests a delayed or impaired transition toward a fully polarized anti-inflammatory state in FOP mice (Fig. 5A).

To further explore FOP-specific transcriptional trajectories, we conducted unbiased k-means clustering on differentially expressed genes (DEGs) between FOP and control cells across subclusters (Table S6), followed by pseudotime trajectory analysis using the Monocle algorithm [45]. This approach enabled us to trace the temporal evolution of gene expression programs during MP differentiation in response to injury. Cells on the tree were ordered from early (monocyte) to late (polarized MPs) states and are colored by pseudotime (Fig. 5B, left panel) or cluster assignment (Fig. 5B right panel).

Using monocytes as the root population, we observed bifurcating trajectories toward pro-inflammatory and anti-inflammatory MPs in both FOP and controls. In line with the subclustering results, control mice exhibited a higher proportion of anti-inflammatory MPs over pseudotime, while FOP mice retained an elevated population of inflammatory MPs, especially at 7 days post-injury (Fig. 5B, right panel).

Focusing on the two divergent terminal branches—representing inflammatory MPs and anti-inflammatory MPs—we performed DEG and GO enrichment analyses, revealing a complex transcriptional landscape (Supplementary Fig. S5A–C, Supplementary Table S7). At 5 days post-injury, both inflammatory and anti-inflammatory MP subclusters in FOP showed upregulation of hypoxia, glycolysis, and pro-inflammatory pathways. Strikingly, by 7 days, the inflammatory branch in FOP mice also exhibited upregulation of oxidative phosphorylation, ECM remodeling, and endochondral ossification pathways—features more typically associated with the anti-inflammatory/remodeling phenotype [46–48]. This suggests that FOP MPs acquire a mixed or transitional state, maintaining inflammatory features while simultaneously engaging in remodeling and ossification-related programs.

To validate these findings, we examined iNOS and CD206 expression—canonical markers of inflammatory and anti-inflammatory polarization, respectively—both *in vivo* and in BMDMs polarized *in vitro*.

At 5 days post-injury FOP mice showed increased number of total MPs (F4/80^+^) (1393.1 ± 68.4 cells/mm^2^ vs 1836.6 ± 164.1 cells/mm^2^, Ctr vs FOP, P < 0.05), of iNOS^+^ cells (166.9 ± 20.1 cells/mm^2^ vs 345.9 ± 17.1 cells/mm^2^, Ctr vs FOP, P < 0.001), and of CD206^+^ cells (971.9 ± 62.4 cells/mm^2^ vs 1547.4 ± 90.3 cells/mm^2^, Ctr vs FOP, P < 0.001) in their muscle as compared to controls. Most interestingly FOP mice also displayed a marked increase in iNOS^+^CD206^+^ double positive cells (68.6 ± 18.1 cells/mm^2^ vs 255.4 ± 39.8 cells/mm^2^, Ctr vs FOP, P < 0.01), supporting the coexistence of inflammatory and remodeling-associated features within the same MP compartment Fig. 5 C-D).

FOP BMDMs subjected to inflammatory polarization showed high iNOS (58 ± 3.9% vs 70 ± 2%, Ctr MPs vs FOP MPs, P < 0.01) expression (Supplementary Fig. S6A-E), but also a moderate yet significant expression of CD206 (25.1 ± 1.8% vs 32.4 ± 4.1%, Ctr MPs vs FOP MPs, P < 0.05) at the protein level compared to controls (Supplementary Fig. S6D-F). Conversely, FOP BMDMs polarized toward an anti-inflammatory phenotype showed elevated CD206 at both mRNA and proteins levels (RNA: 9.4 ± 3.7 vs 20.4 ± 5.5, Ctr MPs vs FOP MPs, P < 0.05) (% CD206^+^ cells: 63.9 ± 12.8% vs 77.6 ± 2.3%, Ctr MPs vs FOP MPs, P < 0.05) (Fig. S6E-F) while simultaneously retaining higher iNOS expression at both RNA and protein levels (RNA: 0.12 ± 0.15 vs 0.49 ± 0.01, Ctr MPs vs FOP MPs, P < 0.05) (% iNOS^+^ cells: 9.3 ± 1.6% vs 22.2 ± 3.3%, Ctr MPs vs FOP MPs, P < 0.01) (Supplementary Fig.S6B-C).

### FOP mice display altered cell–cell interactions during muscle regeneration

Cytokines and growth factors secreted by MPs are critical regulators of the tissue microenvironment and can profoundly influence the fate and behavior of neighbouring mesenchymal cells. As shown before, conditioned media (CM) from FOP BMDPs significantly enhanced FAP osteogenic differentiation *in vitro* (Fig. 1B-C).

**Fig. 6:**
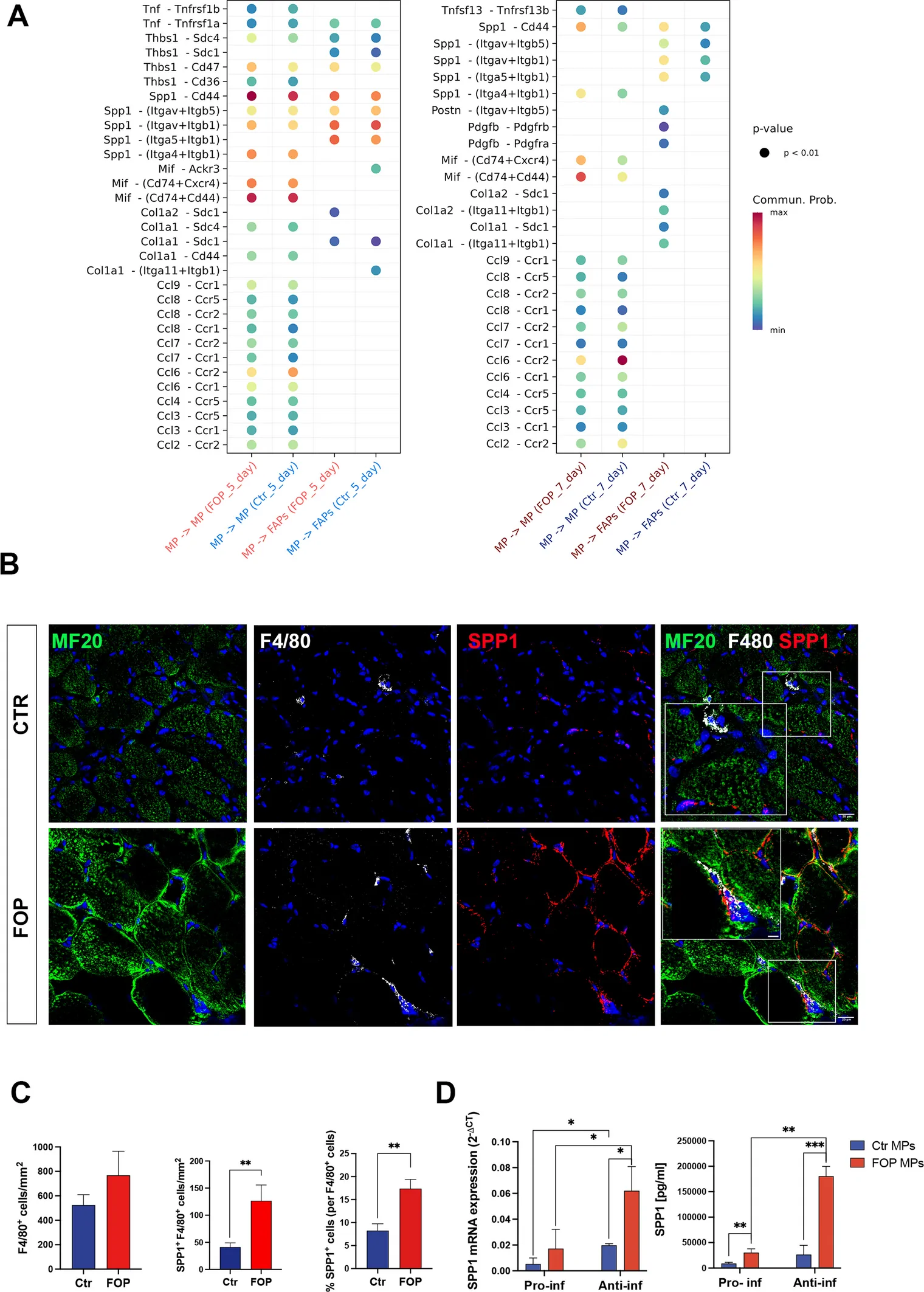
“Analysis of signalling interactions in FAPs and MPs of Ctr and FOP mice after the injury”. A) CellChat bubble plots showing selected upregulated ligand–receptor interactions in the comparison FOP vs Ctr across FOP and control samples at 5 (left panel) and 7 (right panel) days post injury. Each dot represents a ligand–receptor pair, with dot size indicating statistical significance (p < 0.01) and dot color reflecting the computed communication probability. Interactions are grouped by sending and receiving cell populations in MP-to-MP and MP-to-FAP signaling. B) Immunofluorescence staining of gastrocnemius muscle cross-sections of Ctr and FOP mice 5 days post muscle injury using specific antibodies for MF-20 (green, muscle fibers), F4/80 (white, MPs), SPP1 (red). Nuclei were counterstained with DAPI (blue). Scale bar 20 μm. Scale bar 8 μm in magnified images inset. C) Quantification of F4/80^+^ and of F4/80^+^Spp1^+^ cells per square millimetre and frequency (in %) of SPP1^+^ MPs in gastrocnemius muscle cross-sections of FOP and Ctr mice, 7 days after muscle injury (graphs from left to right) (n = 3, with three representative sections of each muscle examined). For F4/80^+^: P = 0.124; for F4/80^+^Spp1.^+^ and %SPP1 statistically significant difference is indicated as **P < 0.01 (two-tailed unpaired Student’s t-test). D) SPP1 expression in Ctr and FOP MPs *in vitro*. Gene expression was investigated by rt-qPCR and protein levels were obtained by ELISA performed on cell supernatant. Data are presented as mean ± SD (n = 3). Statistically significant differences are indicated as *, ** and *** representing P < 0.05, P < 0.01 and P < 0.001, respectively (two-tailed paired and unpaired Student’s t-tests)

To further dissect MP–FAP crosstalk at the lesion site, we performed CellChat analysis on our scRNA-seq dataset, focusing on signaling pathways with MPs as source cells and both MPs and FAPs as targets (Supplementary Table S8−9, Fig. 6A, Supplementary Fig. S7A-B). The analysis identified several communication axes active, mediated by secreted or ECM linked molecules, in both control and FOP mice5 and 7 days post-injury.

Notably, MP-MP communication in FOP revealed a cytokine-rich network that appears to reinforce and perpetuate inflammation. Among the most prominent predicted interactions were TNF–TNFRSF1A, a classic pro-inflammatory axis sustaining NF-κB activation and cytokine release while preventing anti-inflammatory reprogramming [49]; MIF–CD74, associated with MP survival and persistence in chronically inflamed tissues [50]; and CCL2–CCR2, which promotes MPs recruitment and reactivation and is linked to fibrotic pathologies [51] (Fig. 6A, Supplementary Fig. S4A).

In addition to these, the SPP1–CD44 axis stood out for its high predicted interaction probability and dual function: it was detected in MP-MP signaling and also emerged as the dominant MP–FAP interaction in FOP, particularly 7 days post injury. SPP1 is a multifunctional glycophosphoprotein implicated in wound healing, tissue remodeling, and biomineralization, and has been associated with neuromuscular and fibrotic diseases such as Duchenne muscular dystrophy and muscle fibrosis [52–54]. FAPs in FOP early lesions expressed multiple receptors for SPP1, including *Cd44*, *Itgb1*, *Itgb5*, and *Itgav*. Notably, expression of these integrins—beyond *Cd44*—was predominantly observed at day 7 in FOP conditions (Fig. 6A, Supplementary Tables S3, S8-9), suggesting enhanced responsiveness to SPP1 signaling.

CellChat infers ligand–receptor interactions from gene expression as a proxy for protein levels, with inherent limitations. It does not account for post-transcriptional or post-translational regulation, relies on cluster-averaged expression that may mask cellular heterogeneity, and is restricted to curated interaction databases. To experimentally support these predictions, we performed immunofluorescence staining for F4/80 and SPP1 in muscle sections from FOP and control mice, 7 days after pinch injury. FOP mice exhibited both increased MP infiltration (525 ± 84.5 cells/mm^2^ vs 767.9 ± 196.3 cells/mm^2^, Ctr vs FOP, P > 0.05) and a higher proportion of SPP1⁺ MPs as compared to controls, confirming increased SPP1 expression *in vivo* at early stages of lesion formation (41.2 ± 7.8 cells/mm^2^ vs 126.5 ± 29.2 cells/mm^2^, Ctr vs FOP, P < 0.01) (Fig. 6B–C).

We also measured its expression and secretion of SPP1 in FOP BMDMs. RT-qPCR and ELISA confirmed elevated SPP1 mRNA and protein levels, particularly in anti-inflammatory polarized BMDMs (2.8 ± 0.2 vs 9 ± 2.7, Ctr MPs vs FOP MPs, P < 0.05) (26,531.3 ± 18,189 pg/ml vs 180,749.5 ± 18,859.8 pg/ml, Ctr MPs vs FOP MPs, P < 0.001) (Fig. 6D). Interestingly, Activin A *(Inhba)* was not among the differentially expressed genes in FOP MPs, and we detected no significant differences in its expression or in Activin A secretion between FOP and control BMDMs polarized *in vitro* (Supplementary Fig. S5D).

Together, these findings indicate that MPs in FOP establish a self-reinforcing inflammatory network through persistent cytokine signaling. This network not only amplifies their own activation state but also alters the behavior of surrounding progenitor cells. Among the pathways identified, SPP1-associated signaling emerged as a prominent candidate mediator linking the immune and mesenchymal compartments and contributing to the establishment of a permissive microenvironment for pathological ossification.

### SPP1 enhances osteogenic differentiation of FOP FAPs

**Fig. 7:**
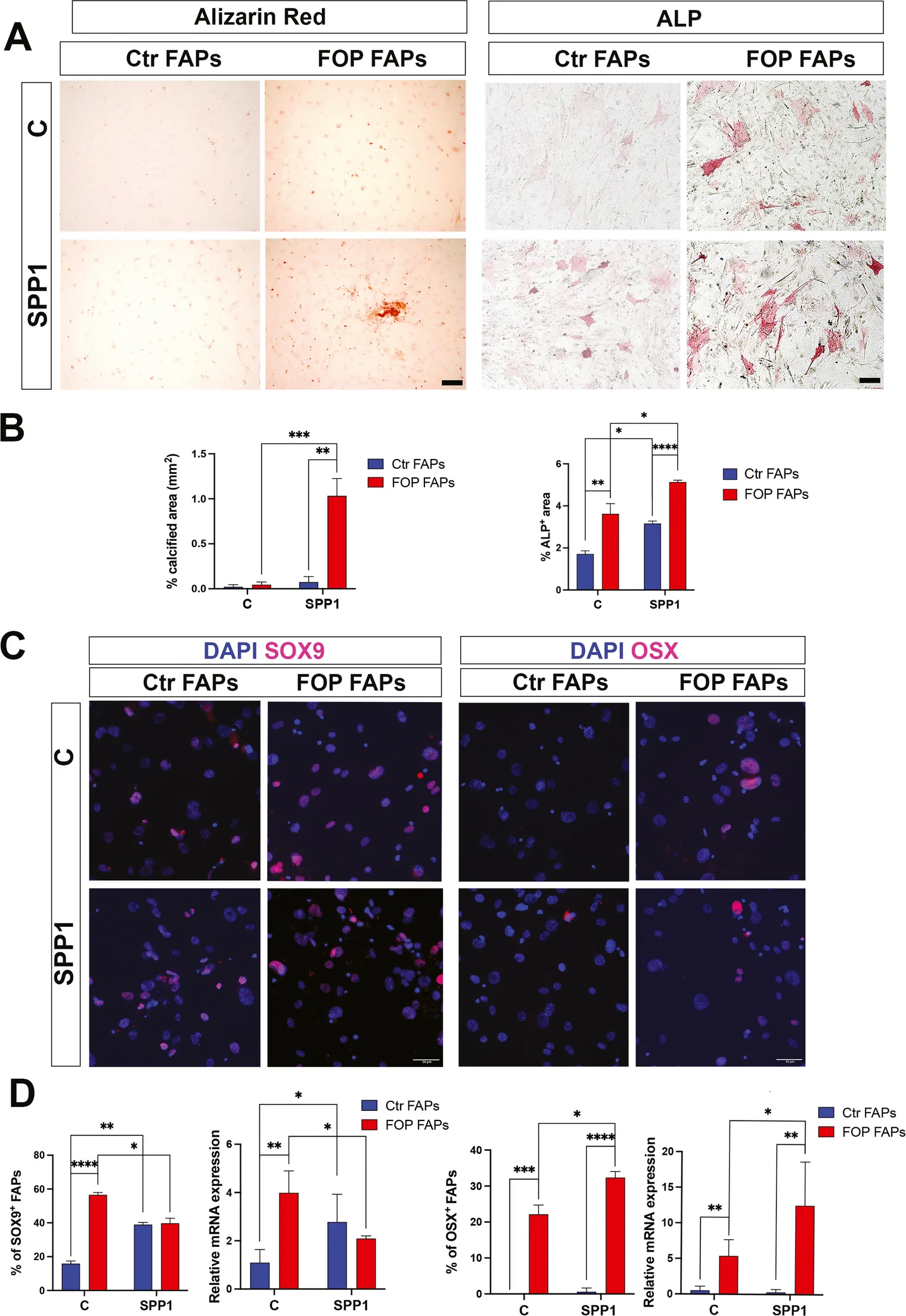
“SPP1 accelerates *in vitro* osteogenesis in FOP FAPs”. A) Ctr and FOP FAPs were maintained in basal conditions (C) or treated with SPP1 (200 ng/ml) for 6 days. and stained for Alizarin Red. (Magnification: 6.3X, Scale Bar: 100 μm) or ALP (Magnification 10X. Scale bar: 100 µm). B) Quantification of area positive for by Alizarin red staining or by ALP staining. Data are presented as mean ± SD (n = 3). Statistically significant differences are indicated as *, **, *** and **** representing P < 0.05, P < 0.01, P < 0.001 and P < 0.0001, respectively. (two-tailed paired and unpaired Student’s t-tests). C) Immunofluorescence staining of Ctr and FOP FAPs 6 days after treatment with SPP1 (200 ng/ml) using specific antibodies against SOX9 (red) or OSX (red). Nuclei were counterstained with DAPI (blue). Scale bar: 50 μm. D) Graphs show the quantification of SOX9^+^ cells or OSX.^+^ cells expressed as percentage, and the relative expression of *Sox9* or *Osx* genes measured by rt-qPCR, using 28S as internal control. Data are presented as mean ± SD (n = 3). Statistically significant differences are indicated as *, ** and **** representing P < 0.05, P < 0.01 and P < 0.0001, respectively (two-tailed paired and unpaired Student’s t-tests)

Based on the results of our bioinformatics analyses, SPP1 was identified as a promising candidate mediator of osteogenic signaling in FOP. To directly assess the functional role of SPP1 in FAP osteogenic priming, we treated FAPs with recombinant SPP1 at 200 ng/ml (the same concentration found to be produced by anti-inflammatory BMDMs polarized *in vitro*) and assessed mineralization after 6 days using Alizarin Red staining. FOP FAPs exhibited a marked increase in total calcified area compared to untreated controls, indicating enhanced osteogenic differentiation (0.04 ± 0.06% vs 1 ± 0.1%, untreated FOP FAPs vs SPP1 FOP FAPs, P < 0.001) (Fig. 7A-B). A similar result was obtained with ALP staining (3.6 ± 0.5% vs 5.1 ± 0.1%, untreated FOP FAPs vs “SPP1” FOP FAPs, P < 0.05) (Fig. 7A-B).

To further dissect the impact of SPP1 on lineage specification, we analyzed the expression of key chondro-osteogenic transcription factors—*Sox9* and *Osx*—via immunofluorescence and rT-qPCR. In control FAPs, SPP1 stimulation led to an upregulation of the expression of *Sox9* (1.1 ± 0.5 vs 2.8 ± 1.1, untreated Ctr FAPs vs SPP1 Ctr FAPs, P < 0.05) ((Fig. 7C-D), while in FOP FAPs, SPP1 selectively enhanced Osx expression (5.3 ± 2.2 vs 12.5 ± 6.1, untreated FOP FAPs vs SPP1 FOP FAPs, P < 0.05) (Fig. 7C-D).

To assess the interaction between SPP1 and canonical ACVR1 signaling, we analyzed early SMAD1/5 phosphorylation in control and FOP FAPs treated with recombinant SPP1 or Activin A for 6 h. While Activin A selectively increased SMAD1/5 phosphorylation in FOP FAPs, (0.91 ± 0.13 vs 1.6 ± 0.30, untreated FOP FAPs vs ActA FOP FAPs, P < 0.01; 0.90 ± 0.05 vs 1.6 ± 0.30, ActA Ctr FAPs vs ActA FOP FAPs, P < 0.05), SPP1 stimulation did not significantly affect SMAD1/5 activation (Supplementary Fig. S7C), supporting the hypothesis that SPP1 acts through parallel pathways rather than directly modulating canonical SMAD1/5 signaling.

These results suggest that SPP1 alone is sufficient to initiate early osteogenic/endochondral commitment programs, even in wild-type cells (Supplementary Figure S7B), though this effect is markedly amplified in FOP FAPs, which are predisposed to this differentiation route.

**Fig. 8:**
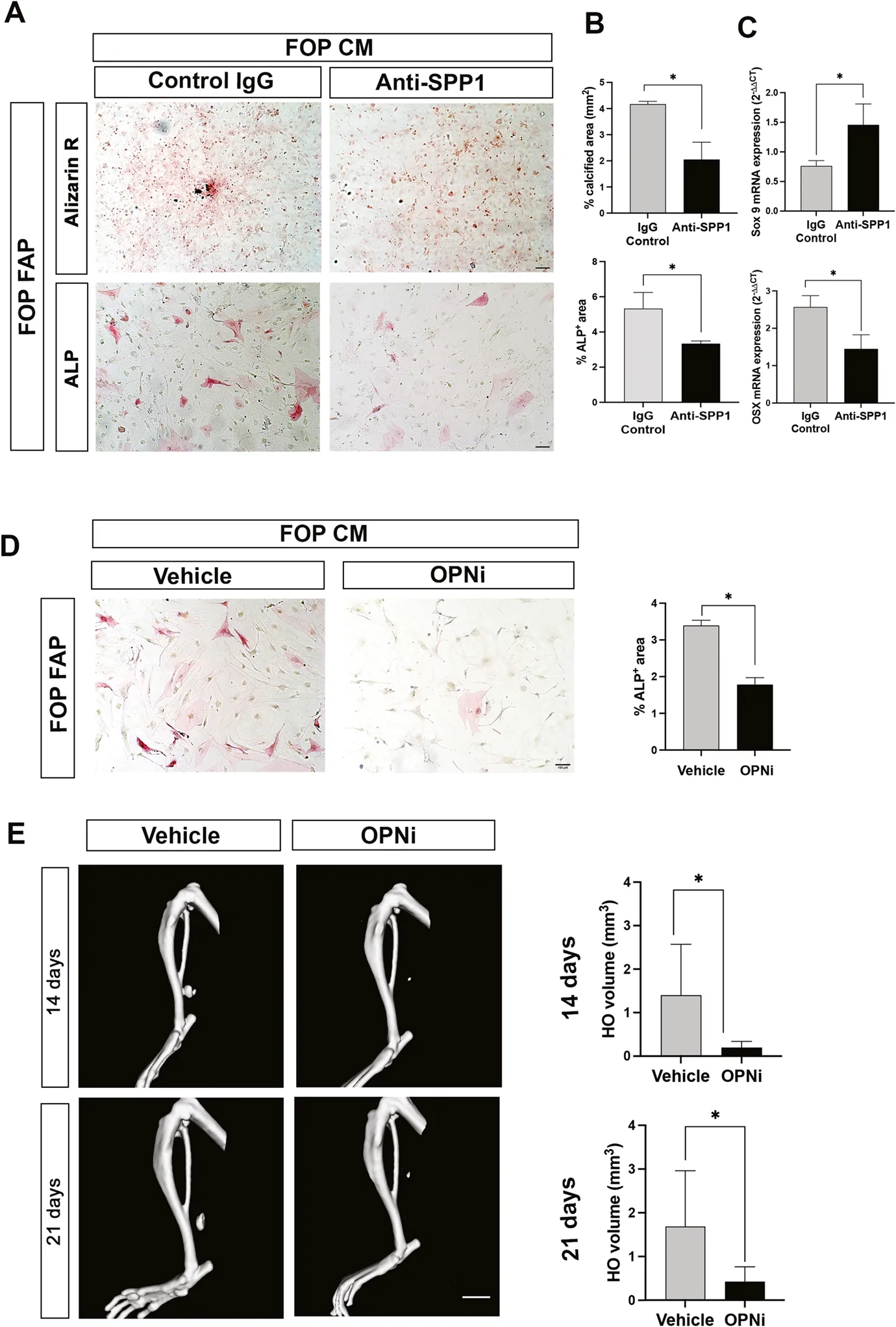
“*In vivo* and *in vitro* effect of SPP1 inhibition on chondro-osteogenic commitment and differentiation”. A) FOP FAPs were treated for 6 days with CM derived from anti-inflammatory polarized FOP MPs in the presence of either anti-SPP1 or control IgG antibody, followed by Alizarin Red or ALP staining. Scale bar: 100 μm. B) Quantification of the Alizarin red^+^ or ALP^+^ area. Data are presented as mean ± SD (n = 3). Statistically significant differences are indicated as * a representing P < 0.05 (two-tailed unpaired Student’s t-test). C) Gene expression levels of chondro/osteogenic markers Sox9 and Osx in FOP FAPs treated with MPs conditioned medium of anti-inflammatory MPs and either anti-SPP1 or control IgG antibody. Results are normalized to 28S gene used as internal control. Data are presented as mean ± SD (n = 3). Statistically significant differences are indicated as * representing P < 0.05, (two-tailed paired and unpaired Student’s t-tests). D) FOP FAPs were treated for 6 days with CM derived from anti-inflammatory polarized FOP MPs in the presence of the OPNi inhibitor (25 μg/ml) or DMSO (Vehicle), followed by ALP staining. Graphs show the relative quantification of ALP + area. Data are presented as mean ± SD (n = 3). Statistical significance is indicated as *P < 0.05 (two-tailed paired and unpaired Student’s t-tests). Scale bar: 100 μm. E) Micro-CT of injured FOP mice treated with OPNi or Vehicle at 14 and 21 days post injury. Graphs represent the relative quantification of HO volume. Data are presented as mean ± SD (n = 5). Statistically significant differences are indicated as * representing P < 0.05 (two-tailed unpaired Student’s t-test). Scale bar: 5 mm

Given our previous findings showing that CM from polarized FOP BMDMs enhances osteogenic commitment in FOP FAPs (Fig. 1C), we sought to determine whether SPP1 was a major mediator of this effect. To this end, we used a specific monoclonal antibody for SPP1 to neutralize SPP1 signaling [55] (Supplementary Fig.S8A) in FAPs cultured with BMDM-derived CM. In Ctr or FOP FAPs treated with CM from control BMDMs, no changes in Alizarin Red staining were observed upon antibody treatment (Supplementary Fig. 8E-F). However, the ability of CM from FOP BMDMs to promote FAP osteogenic commitment was impaired in presence of the SPP1-blocking antibody (Fig. 8A-C, Supplementary Fig. 8B-D). Alizarin Red staining revealed a significant reduction when FOP FAPs were treated with CM from FOP BMDMs in the presence of the SPP1-blocking antibody (4.2 ± 0.1% vs 2 ± 0.6%, control IgG vs Anti-SPP1, P < 0.05) (Fig. 8B-C). Similarly, ALP staining was reduced in FOP FAPs treated with CM from FOP BMDMs in the presence of the SPP1-blocking antibody (5.3 ± 0.9% vs 3.3 ± 0.1%, IgG Control vs αSPP1, P < 0.05).

Furthermore, under these conditions, SPP1 blockade led to increased *Sox9* expression (0.8 ± 0.1 vs 1.4 ± 0.3, control IgG vs Anti-SPP1, P < 0.05) and decreased *Osx* expression (2.6 ± 0.3 vs 1.4 ± 0.4, control IgG vs Anti-SPP1, P < 0.05) as compared to cells treated with control IgG (Fig. 8C), suggesting a partial shift away from osteogenic committment toward a less differentiated or more chondrogenic-like state.

To further validate the effect of SPP1 inhibition, we used a specific pharmacological inhibitor of SPP1 expression that we recently validated in a model of muscle fibrosis [26]. Consistently, inhibition of SPP1 using OPNi in FOP FAPs treated with CM from FOP BMDMs also led to a significant reduction in osteogenic committment, as measured by ALP staining (3.4 ± 0.1% vs 1.8 ± 0.2%, Vehicle vs OPNi, P < 0.05) (Fig. 8D).

Finally, we assessed whether inhibition of SPP1 could attenuate HO formation *in vivo* in FOP mice. OPNi was administered to FOP mice by IP injection—starting one day before injury and every 3 days thereafter. A control group received vehicle injections, and µCT analyses were performed at 14 and 21 days post-injury. HO volume was quantified in vehicle- and OPNi-treated FOP mice processed in parallel within the same experimental cohort, thereby accounting for inter-experimental variability in HO severity.

The analysis revealed a significant reduction in ectopic bone volume in OPNi-treated mice compared to controls at both 14 and 21 days post-injury (1.4 ± 1.2 mm^3^ vs 0.2 ± 0.1 mm^3^, Vehicle vs OPNi, P < 0.05, 14 days; 1.7 ± 1.3 mm^3^ vs 0.4 ± 0.3 mm^3^, Vehicle vs OPNi, P < 0.05, 21 days (Fig. 8E).

Taken together, these findings support a role for SPP1 in modulating MP–FAP crosstalk and promoting osteogenic commitment in FOP. Although SPP1 inhibition did not completely abolish HO formation, both the *in vitro* and *in vivo* data indicate that SPP1 contributes functionally to the establishment of the pro-osteogenic microenvironment underlying pathological ossification in FOP.

## Discussion

Our study highlights the early role of MPs in influencing the local environment that leads to HO in FOP. While FAPs are recognized as the main mesenchymal drivers of ectopic bone formation, our results show that their differentiation potential is strongly influenced by signals from the immune compartment. We identify SPP1 as a MP-secreted factor that promotes osteogenic differentiation of FOP FAPs and may act as an important mediator significantly contributing to the etiologic link between inflammation and pathological osteogenic commitment in FOP.

Using single-cell RNA sequencing (scRNA-seq), we observed a significant increase of both FAPs and MPs in FOP mice after injury, suggesting that these two populations dominate the early phase of ectopic endochondral ossification. DEG and GO analyses revealed that FOP FAPs upregulate several genes associated with a profibrotic and osteogenic response following muscle injury. Notably, *Acta2*, *Fn1*, and *Timp1* were significantly overexpressed, suggesting activation of a fibrotic program. In parallel, genes involved in ECM remodeling—such as *Sparc*, *Postn*, and *Tagln*—were also upregulated, indicating a shift toward a tissue-remodeling phenotype. Osteochondral transcription factors including Sox9 and Runx2 were elevated, reflecting the increased propensity toward endochondral ossification as compared to controls [17].

Importantly, FOP FAPs exhibited increased sensitivity to inflammatory stimuli making them particularly susceptible to MP-derived cytokines and growth factors, which are abundantly secreted during the early inflammatory phase of HO. These signals not only sustain local inflammation but also reinforce the osteogenic program in FAPs, thus contributing to the establishment of a feed-forward loop that drives ectopic ossification [56].

MP depletion via clodronate led to a marked reduction in ectopic bone formation, supporting the idea that these cells are critically involved in HO progression. Interestingly, FOP MPs exhibited a transcriptional profile that was not easily classifiable into conventional inflammatory or anti-inflammatory categories. Alongside classical inflammatory cytokines and NF-κB activation, we found that MPs show signatures associated with ECM remodeling, oxidative phosphorylation, and even osteoclast-related genes such as *Acp5* and cathepsins, indicating a mixed polarization state.

This plasticity likely reflects the altered tissue environment of FOP HO sites, which are characterized by persistent inflammation, local hypoxia, and altered mechanical stimuli. Similar context-dependent MP phenotypes have been described in fibrotic and neoplastic conditions, where MPs actively influence disease progression and tissue remodeling [14, 41, 48]. In this context, our analyses provide a detailed characterization of the transcriptional programs associated with MPs during the early stages of FOP lesion formation. Our pseudotime analysis shows that FOP MPs follow a delayed or altered differentiation trajectory, maintaining inflammatory features while activating tissue remodeling programs. In addition, the persistent expression of both iNOS and CD206 in FOP mice *in vivo* and in FOP BMDMs polarized *in vitro* under classically inflammatory and anti-inflammatory conditions, respectively, suggests a “shapeshifting” polarization state that diverts from canonical paradigms, potentially reflecting MP adaptation to the complex early HO microenvironment, where inflammatory, profibrotic, and tissue-remodeling cues coexist and may contribute to aberrant skeletogenic progression. Importantly, the relevance of these observations lies not simply in the expression of individual inflammatory or remodeling-associated markers, but in the coordinated emergence of these transcriptional programs during the earliest stages of lesion development, concomitant with stromal chondro-osteogenic commitment and prior to overt HO formation. The relative contribution of tissue-resident and infiltrating MP populations remains unclear in this context, and more specific phenotypic and functional analyses, potentially including alternative models such as CCR2^−/−^ mice. Nevertheless, the coexistence of inflammatory and remodeling programs observed in FOP MPs highlights the complexity of the early HO microenvironment and suggests the involvement of multiple MP states during lesion initiation.

Among the secreted factors upregulated in FOP MPs, SPP1 emerged as a strong candidate for mediating immune–mesenchymal crosstalk. We show that SPP1 is highly expressed by FOP MPs, particularly in those polarized with anti-inflammatory cues, and that its presence is sufficient to strongly enhance osteogenic differentiation of FOP FAPs *in vitro*. Blocking SPP1 with a neutralizing antibody or a pharmacological inhibitor led to partial reversion of the osteogenic programs *in vitro*, while *in vivo* treatment with OPNi reduced HO formation.

SPP1 has been extensively implicated in cancer biology, inflammatory disorders, and fibrotic diseases, where it promotes cytokine production, tissue remodeling, and fibrosis through interactions with integrins and CD44 [57, 58]. Consistently, suppression of SPP1 signaling reduces cardiac fibrosis [59, 60], EndoMT in injured skeletal muscle [26] and traumatic HO [61, 62]. Moreover, we observed upregulation of *Mmp14* in FOP MPs and several additional *MMPs* in FOP FAPs (Tables S2−3). MMP14 (MT1-MMP) is involved in matrix remodeling and activation of latent TGF-β [63, 64], and its deficiency impairs endochondral ossification *in vivo* [50, 65]. Interestingly, a recent report described an FOP patient with an unusually mild HO phenotype associated with MMP9 deficiency [66]. Although *Mmp9* was not differentially expressed in our dataset and the mechanistic relationship between SPP1 and MMP activity remains unclear, the coordinated upregulation of remodeling-associated pathways further supports the activation of tissue-remodeling programs during early HO initiation.

These findings further support the contribution of inflammatory microenvironmental signals to FOP pathogenesis. The seminal identification of Activin A as a signaling ligand for mutant ACVR1 established a critical mechanistic link between inflammation and HO formation in FOP, since Activin A is abundantly produced by immune cells during inflammatory responses [3, 67]. Beyond its pathological role in FOP, accumulating evidence also supports broader functions of Activin A in skeletal biology, including mesenchymal chondrogenic and osteogenic differentiation, fracture repair, and non-genetic HO formation [68–71]. Consistently, recent studies using distinct mutant ACVR1-driven HO models further highlighted the contribution of MP-associated Activin A signaling during injury-associated HO formation and tissue repair responses [72]. In the context of FOP, however, mutant ACVR1 aberrantly interprets Activin A signaling, thereby promoting ectopic chondro-osteogenic differentiation and endochondral ossification [3, 67].

Our data now indicate that immune cells can influence FAP differentiation through additional, parallel mechanisms such as signaling via SPP1/CD44 or SPP1/integrins, which do not appear to directly activate SMAD phosphorylation. These findings further support the complexity of the signaling networks driving early HO initiation in FOP and may explain the partial efficacy of single-target therapies in clinical trials [9, 73].

Although SPP1 inhibition significantly attenuated osteogenic commitment and HO formation, the effects were partial, suggesting that additional inflammatory and stromal-derived signals likely cooperate in shaping the pro-skeletogenic niche in FOP lesions. Indeed, our transcriptional analyses identified activation of multiple pathways associated with inflammation, ECM remodeling, hypoxia responses, and TGF-β signaling. Previous studies have shown that cytokines such as IL6, IL1β and TGF-β, together with ECM-derived cues, can profoundly influence mesenchymal progenitor fate and pathological osteogenic differentiation [27, 74–76]. Together, these findings suggest that SPP1 acts within a broader inflammatory and tissue-remodeling signaling network driving early HO initiation.

Further studies will be needed to better define the functional role of SPP1 in FOP and to explore its potential as a therapeutic target. Given the broad and context-dependent functions of SPP1 in different tissues, it will be important to understand whether systemic inhibition is a viable approach or if more selective strategies—targeting specific cell types or using local delivery—are required. In this work, we focused mainly on the interaction between MPs and FAPs, but other immune or stromal cells present in the lesion environment could also contribute to disease progression and should be considered in future studies.

In summary, our findings support a model in which MPs help shape a microenvironment that favors ectopic bone formation in FOP, through sustained inflammatory signaling and secretion of matrix-remodeling factors. Among these, SPP1 emerges as an important contributor to enhanced FAP osteogenic fate. Modulating this immune–mesenchymal crosstalk could provide an additional therapeutic strategy, potentially complementing ActA/ACVR1-targeted treatments, especially in the early, inflammation-driven stages of ectopic bone development.

## Supplementary Information

Below is the link to the electronic supplementary material.

Supplementary file1 (XLSX 649 KB)

Supplementary file2 (XLSX 496 KB)

Supplementary file3 (XLSX 527 KB)

Supplementary file4 (XLSX 497 KB)

Supplementary file5 (XLSX 352 KB)

Supplementary file6 (XLSX 337 KB)

Supplementary file7 (XLSX 104 KB)

Supplementary file8 (XLSX 72 KB)

Supplementary file9 (XLSX 123 KB)

Supplementary file10 (PDF 1699 KB)

## Data Availability

The single-cell RNA sequencing data generated in this study have been deposited in the GEO database under the accession code GSE302734.
