# Supplementary material for: Single-Cell dissection of fibrodysplasia ossificans progressiva identifies SPP1 as a mediator of macrophage–fibroadipogenic progenitors crosstalk
Source: Cell Mol Life Sci. 2026 Jul 2;83(1):344. doi: 10.1007/s00018-026-06320-z (PMC13612808; doi:10.1007/s00018-026-06320-z)
Supplement: Supplementary file 1 [file 18_2026_6320_MOESM10_ESM.pdf]

Fig. S1

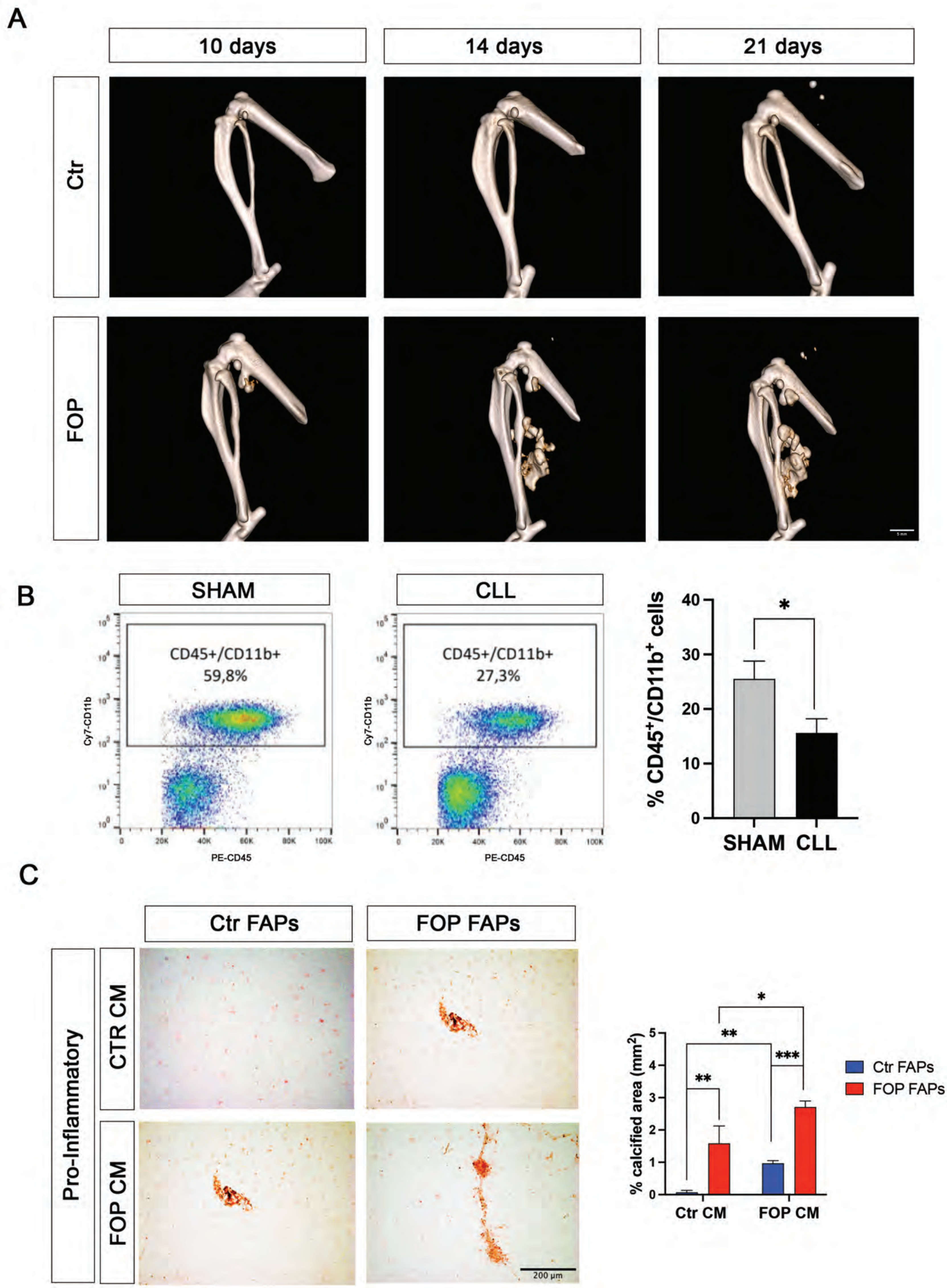
A
10 days
14 days
21 days
Ctr
FOP
B
SHAM
CLL
CD45+/CD11b+
59,8%
27,3%
Cy7-CD11b
PE-CD45
% CD45+/CD11b+ cells
SHAM
CLL
C
Ctr FAPs
FOP FAPs
Pro-Inflammatory
CTR CM
FOP CM
200 µm
% calcified area (mm2)
Ctr CM
FOP CM
Ctr FAPs
FOP FAPs

Fig. S2

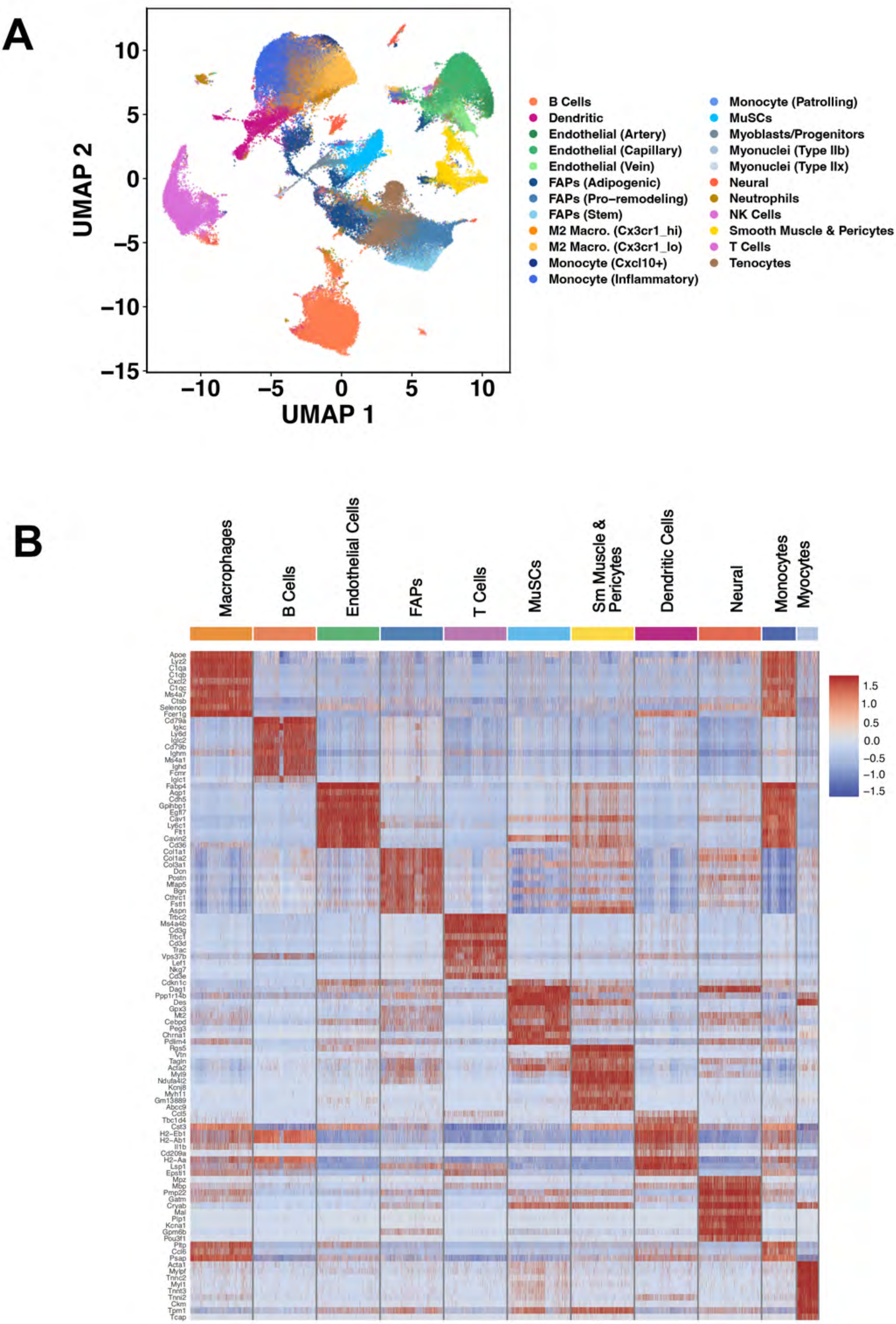
A
UMAP 1
UMAP 2
−10
−5
0
5
10
−15
B Cells
Dendritic
Endothelial (Artery)
Endothelial (Capillary)
Endothelial (Vein)
FAPs (Adipogenic)
FAPs (Pro−remodeling)
FAPs (Stem)
M2 Macro. (Cx3cr1_hi)
M2 Macro. (Cx3cr1_lo)
Monocyte (Cxcl10+)
Monocyte (Inflammatory)
Monocyte (Patrolling)
MuSCs
Myoblasts/Progenitors
Myonuclei (Type IIb)
Myonuclei (Type IIx)
Neural
Neutrophils
NK Cells
Smooth Muscle & Pericytes
T Cells
Tenocytes
B
Macrophages
B Cells
Endothelial Cells
FAPs
T Cells
MuSCs
Sm Muscle & Pericytes
Dendritic Cells
Neural
Monocytes
Myocytes
1.5
1.0
0.5
0.0
−0.5
−1.0
−1.5
Apoe
Lyz2
C1qa
C1qb
Cxcl2
C1qc
Ms4a7
Ctsb
Selenop
Fcer1g
Cd79a
Igkc
Ly6d
Iglc2
Cd79b
Ighm
Ms4a1
Ighd
Fcmr
Iglc1
Fabp4
Aqp1
Cdh5
Gpihbp1
Egfl7
Cav1
Ly6c1
Flt1
Cavin2
Cd36
Col1a1
Col1a2
Col3a1
Dcn
Postn
Mfap5
Bgn
Cthrc1
Fstl1
Aspn
Trbc2
Ms4a4b
Cd3g
Trbc1
Cd3d
Trac
Vps37b
Lef1
Nkg7
Cd3e
Cdkn1c
Dag1
Ppp1r14b
Des
Gpx3
Mt2
Cebpd
Peg3
Chrna1
Pdlim4
Rgs5
Vtn
Tagln
Acta2
Myl9
Ndufa4l2
Kcnj8
Myh11
Gm13889
Abcc9
Ccl5
Tbc1d4
Cst3
H2−Eb1
H2−Ab1
Il1b
Cd209a
H2−Aa
Lsp1
Epsti1
Mpz
Mbp
Pmp22
Gatm
Cryab
Mal
Plp1
Kcna1
Gpm6b
Pou3f1
Pltp
Ccl6
Psap
Acta1
Mylpf
Tnnc2
Myl1
Tnnt3
Tnni2
Ckm
Tpm1
Tcap

Fig. S3

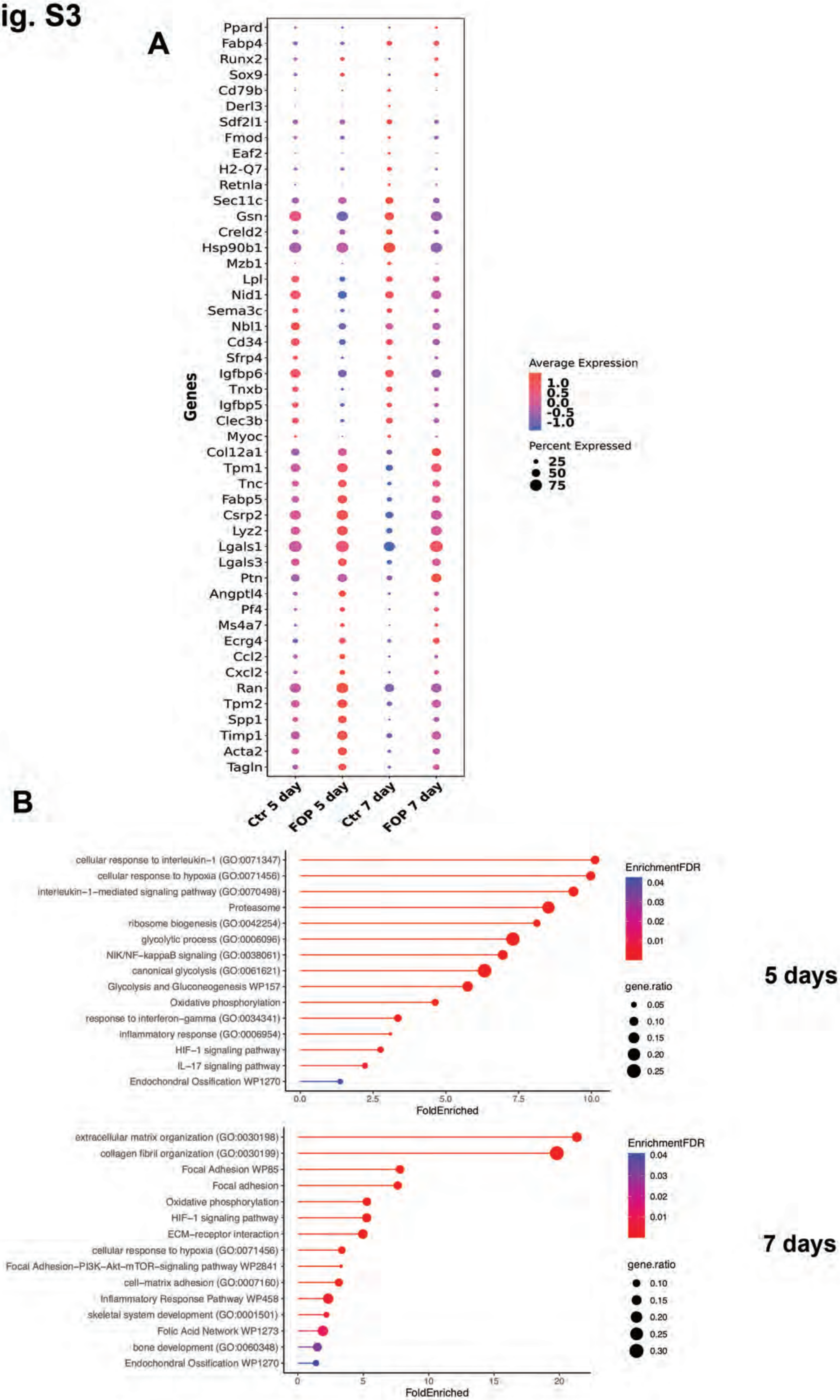

A
Ppard
Fabp4
Runx2
Sox9
Cd79b
Derl3
Sdf2l1
Fmod
Eaf2
H2-Q7
Retnla
Sec11c
Gsn
Creld2
Hsp90b1
Mzb1
Lpl
Nid1
Sema3c
Nbl1
Cd34
Sfrp4
Igfbp6
Tnxb
Igfbp5
Clec3b
Myoc
Col12a1
Tpm1
Tnc
Fabp5
Csrp2
Lyz2
Lgals1
Lgals3
Ptn
Angptl4
Pf4
Ms4a7
Ecrg4
Ccl2
Cxcl2
Ran
Tpm2
Spp1
Timp1
Acta2
Tagln
Genes
Ctr 5 day
FOP 5 day
Ctr 7 day
FOP 7 day
Average Expression
1.0
0.5
0.0
-0.5
-1.0
Percent Expressed
25
50
75
B
cellular response to interleukin-1 (GO:0071347)
cellular response to hypoxia (GO:0071456)
interleukin-1-mediated signaling pathway (GO:0070498)
Proteasome
ribosome biogenesis (GO:0042254)
glycolytic process (GO:0006096)
NIK/NF-kappaB signaling (GO:0038061)
canonical glycolysis (GO:0061621)
Glycolysis and Gluconeogenesis WP157
Oxidative phosphorylation
response to interferon-gamma (GO:0034341)
inflammatory response (GO:0006954)
HIF-1 signaling pathway
IL-17 signaling pathway
Endochondral Ossification WP1270
0.0
2.5
5.0
7.5
10.0
FoldEnriched
EnrichmentFDR
0.04
0.03
0.02
0.01
gene.ratio
0.05
0.10
0.15
0.20
0.25
5 days
extracellular matrix organization (GO:0030198)
collagen fibril organization (GO:0030199)
Focal Adhesion WP85
Focal adhesion
Oxidative phosphorylation
HIF-1 signaling pathway
ECM-receptor interaction
cellular response to hypoxia (GO:0071456)
Focal Adhesion-PI3K-Akt-mTOR-signaling pathway WP2841
cell-matrix adhesion (GO:0007160)
Inflammatory Response Pathway WP458
skeletal system development (GO:0001501)
Folic Acid Network WP1273
bone development (GO:0060348)
Endochondral Ossification WP1270
0
5
10
15
20
FoldEnriched
EnrichmentFDR
0.04
0.03
0.02
0.01
gene.ratio
0.10
0.15
0.20
0.25
0.30
7 days

Fig. S4

A

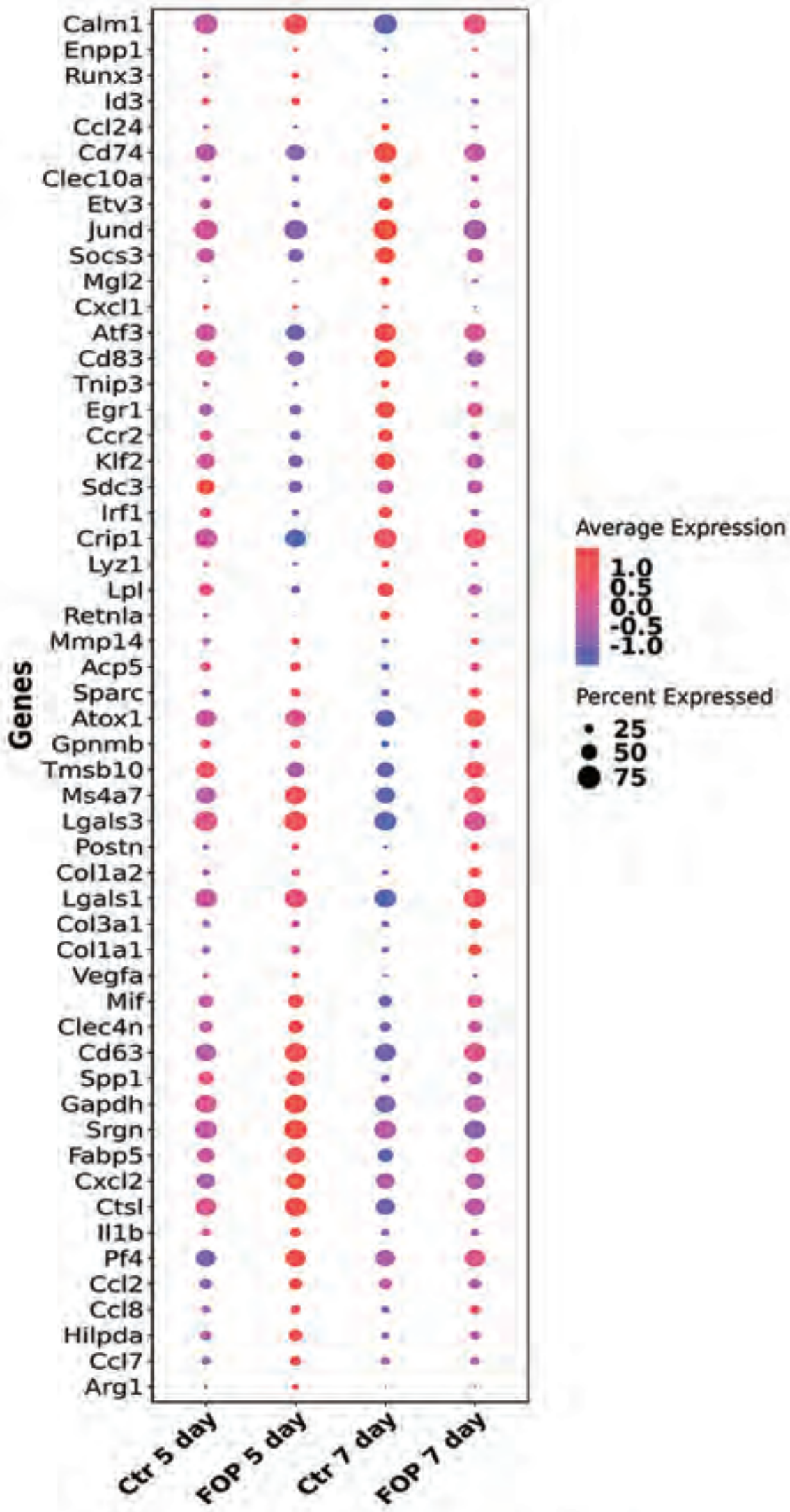
Calm1
Enpp1
Runx3
Id3
Ccl24
Cd74
Clec10a
Etv3
Jund
Socs3
Mgl2
Cxcl1
Atf3
Cd83
Tnip3
Egr1
Ccr2
Klf2
Sdc3
Irf1
Crip1
Lyz1
Lpl
Retnla
Mmp14
Acp5
Sparc
Atox1
Gpnmb
Tmsb10
Ms4a7
Lgals3
Postn
Col1a2
Lgals1
Col3a1
Col1a1
Vegfa
Mif
Clec4n
Cd63
Spp1
Gapdh
Srgn
Fabp5
Cxcl2
Ctsl
Il1b
Pf4
Ccl2
Ccl8
Hilpda
Ccl7
Arg1
Genes
Ctr 5 day
FOP 5 day
Ctr 7 day
FOP 7 day
Average Expression
1.0
0.5
0.0
-0.5
-1.0
Percent Expressed
25
50
75

B

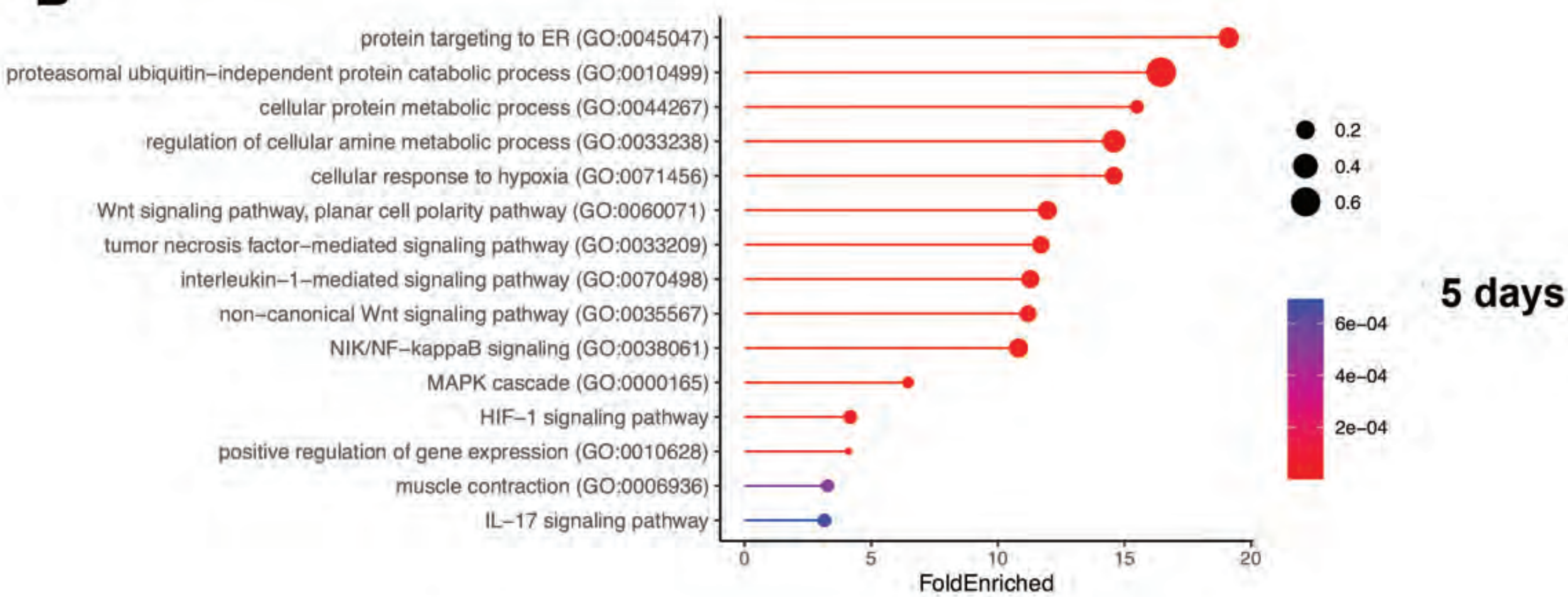
protein targeting to ER (GO:0045047)
proteasomal ubiquitin–independent protein catabolic process (GO:0010499)
cellular protein metabolic process (GO:0044267)
regulation of cellular amine metabolic process (GO:0033238)
cellular response to hypoxia (GO:0071456)
Wnt signaling pathway, planar cell polarity pathway (GO:0060071)
tumor necrosis factor–mediated signaling pathway (GO:0033209)
interleukin–1–mediated signaling pathway (GO:0070498)
non–canonical Wnt signaling pathway (GO:0035567)
NIK/NF–kappaB signaling (GO:0038061)
MAPK cascade (GO:0000165)
HIF–1 signaling pathway
positive regulation of gene expression (GO:0010628)
muscle contraction (GO:0006936)
IL–17 signaling pathway
0
5
10
15
20
FoldEnriched
0.2
0.4
0.6
6e–04
4e–04
2e–04
5 days

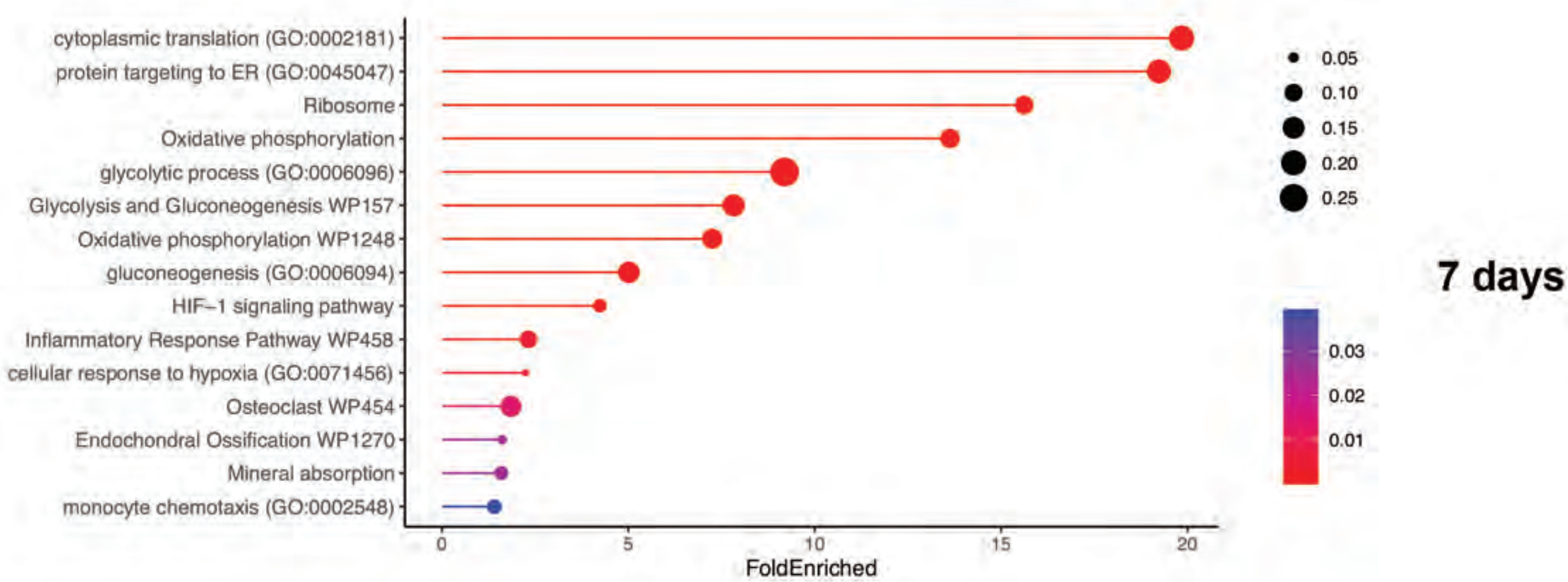
cytoplasmic translation (GO:0002181)
protein targeting to ER (GO:0045047)
Ribosome
Oxidative phosphorylation
glycolytic process (GO:0006096)
Glycolysis and Gluconeogenesis WP157
Oxidative phosphorylation WP1248
gluconeogenesis (GO:0006094)
HIF–1 signaling pathway
Inflammatory Response Pathway WP458
cellular response to hypoxia (GO:0071456)
Osteoclast WP454
Endochondral Ossification WP1270
Mineral absorption
monocyte chemotaxis (GO:0002548)
0
5
10
15
20
FoldEnriched
0.05
0.10
0.15
0.20
0.25
0.03
0.02
0.01
7 days

Fig. S5

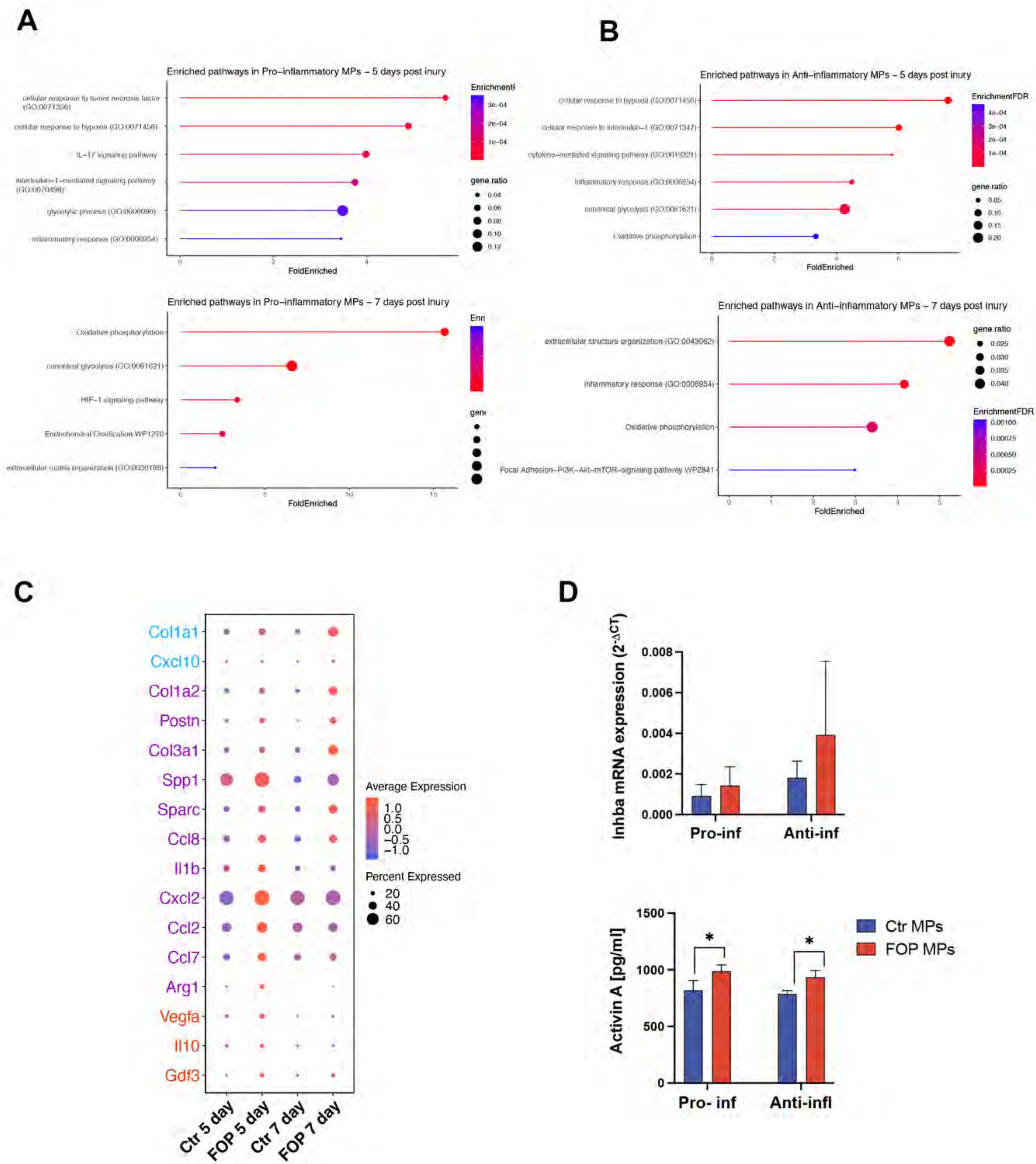
A
Enriched pathways in Pro−inflammatory MPs − 5 days post inury
cellular response to tumor necrosis factor (GO:0071356)
cellular response to hypoxia (GO:0071456)
IL−17 signaling pathway
interleukin−1−mediated signaling pathway (GO:0070498)
glycolytic process (GO:0006096)
inflammatory response (GO:0006954)
FoldEnriched
gene.ratio
0.04
0.06
0.08
0.10
0.12
3e−04
2e−04
1e−04
Enriched pathways in Pro−inflammatory MPs − 7 days post inury
Oxidative phosphorylation
canonical glycolysis (GO:0061621)
HIF−1 signaling pathway
Endochondral Ossification WP1270
extracellular matrix organization (GO:0030198)
FoldEnriched
B
Enriched pathways in Anti−inflammatory MPs − 5 days post inury
cellular response to hypoxia (GO:0071456)
cellular response to interleukin−1 (GO:0071347)
cytokine−mediated signaling pathway (GO:0019221)
inflammatory response (GO:0006954)
canonical glycolysis (GO:0061621)
Oxidative phosphorylation
EnrichmentFDR
4e−04
3e−04
2e−04
1e−04
gene.ratio
0.05
0.10
0.15
0.20
FoldEnriched
Enriched pathways in Anti−inflammatory MPs − 7 days post inury
extracellular structure organization (GO:0043062)
inflammatory response (GO:0006954)
Oxidative phosphorylation
Focal Adhesion−PI3K−Akt−mTOR−signaling pathway WP2841
gene.ratio
0.025
0.030
0.035
0.040
EnrichmentFDR
0.00100
0.00075
0.00050
0.00025
FoldEnriched
C
Col1a1
Cxcl10
Col1a2
Postn
Col3a1
Spp1
Sparc
Ccl8
Il1b
Cxcl2
Ccl2
Ccl7
Arg1
Vegfa
Il10
Gdf3
Average Expression
1.0
0.5
0.0
−0.5
−1.0
Percent Expressed
20
40
60
Ctr 5 day
FOP 5 day
Ctr 7 day
FOP 7 day
D
Inhba mRNA expression (2^-ΔCT)
0.008
0.006
0.004
0.002
0.000
Pro-inf
Anti-inf
Activin A [pg/ml]
1500
1000
500
0
*
*
Pro- inf
Anti-infl
Ctr MPs
FOP MPs

Fig. S6

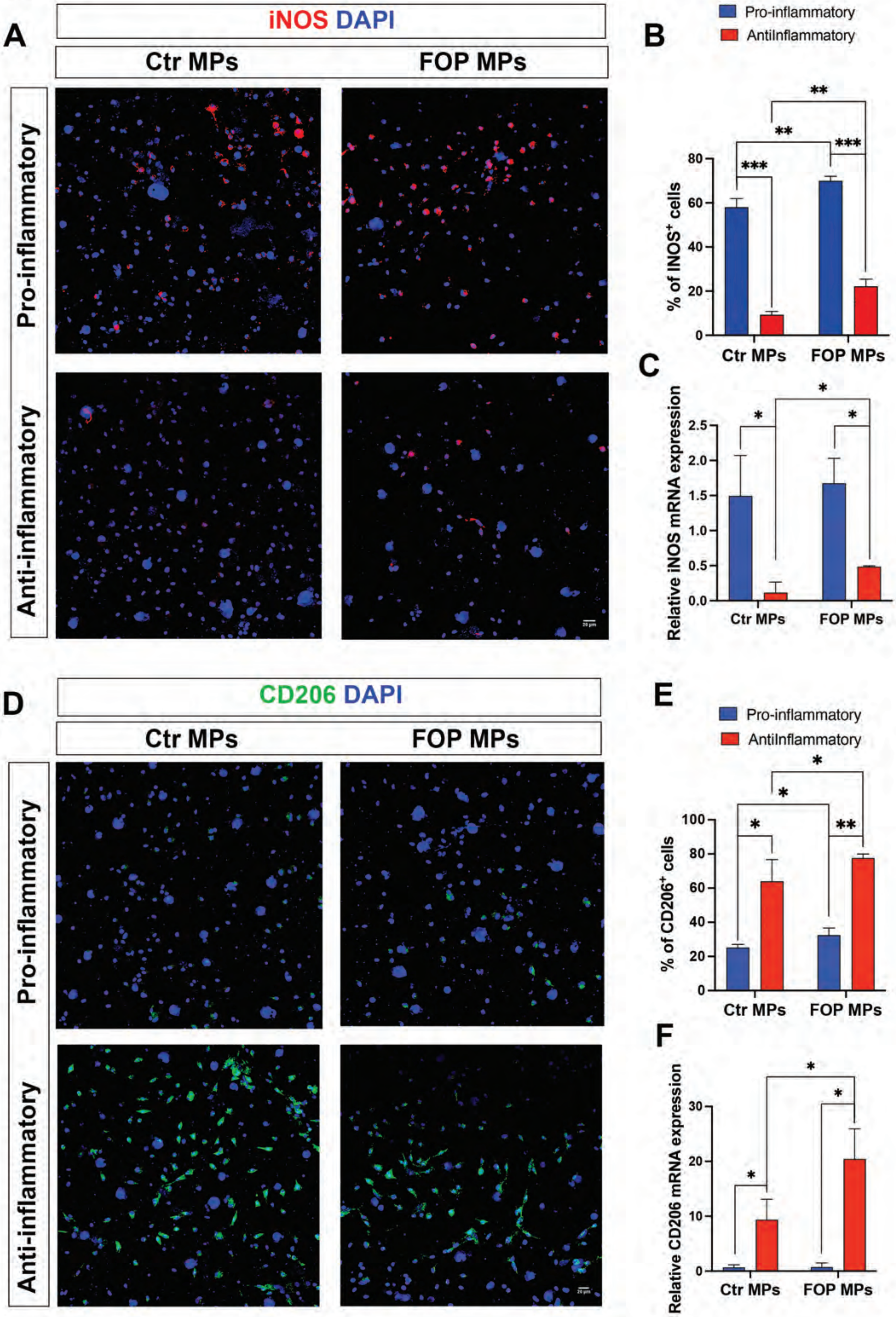
A
iNOS DAPI
Ctr MPs
FOP MPs
Pro-inflammatory
Anti-inflammatory
B
Pro-inflammatory
AntiInflammatory
% of iNOS+ cells
0
20
40
60
80
**
**
***
***
Ctr MPs
FOP MPs
C
Relative iNOS mRNA expression
0.0
0.5
1.0
1.5
2.0
2.5
*
*
*
Ctr MPs
FOP MPs
D
CD206 DAPI
Ctr MPs
FOP MPs
Pro-inflammatory
Anti-inflammatory
E
Pro-inflammatory
AntiInflammatory
% of CD206+ cells
0
20
40
60
80
100
*
*
*
**
Ctr MPs
FOP MPs
F
Relative CD206 mRNA expression
0
10
20
30
*
*
*
Ctr MPs
FOP MPs

Fig. S7

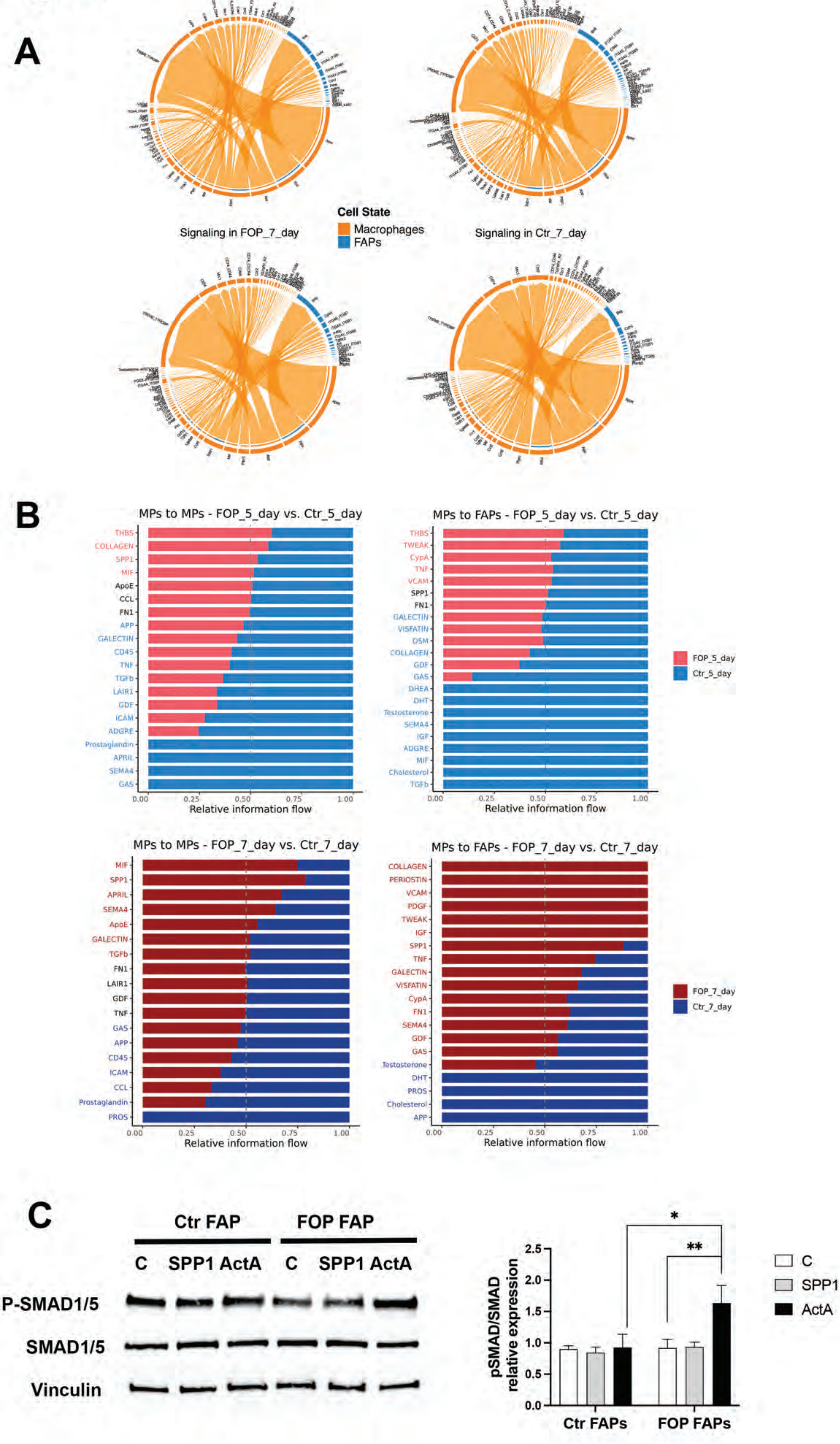
A
Signaling in FOP_5_day
Signaling in Ctr_5_day
Signaling in FOP_7_day
Signaling in Ctr_7_day
Cell State
Macrophages
FAPs
B
MPs to MPs - FOP_5_day vs. Ctr_5_day
THBS
COLLAGEN
SPP1
MIF
ApoE
CCL
FN1
APP
GALECTIN
CD45
TNF
TGFb
LAIR1
GDF
ICAM
ADGRE
Prostaglandin
APRIL
SEMA4
GAS
0.00 0.25 0.50 0.75 1.00
Relative information flow
MPs to FAPs - FOP_5_day vs. Ctr_5_day
THBS
TWEAK
CypA
TNF
VCAM
SPP1
FN1
GALECTIN
VISFATIN
OSM
COLLAGEN
GDF
GAS
DHEA
DHT
Testosterone
SEMA4
IGF
ADGRE
MIF
Cholesterol
TGFb
0.00 0.25 0.50 0.75 1.00
Relative information flow
FOP_5_day
Ctr_5_day
MPs to MPs - FOP_7_day vs. Ctr_7_day
MIF
SPP1
APRIL
SEMA4
ApoE
GALECTIN
TGFb
FN1
LAIR1
GDF
TNF
GAS
APP
CD45
ICAM
CCL
Prostaglandin
PROS
0.00 0.25 0.50 0.75 1.00
Relative information flow
MPs to FAPs - FOP_7_day vs. Ctr_7_day
COLLAGEN
PERIOSTIN
VCAM
PDGF
TWEAK
IGF
SPP1
TNF
GALECTIN
VISFATIN
CypA
FN1
SEMA4
GDF
GAS
Testosterone
DHT
PROS
Cholesterol
APP
0.00 0.25 0.50 0.75 1.00
Relative information flow
FOP_7_day
Ctr_7_day
C
Ctr FAP
FOP FAP
C SPP1 ActA C SPP1 ActA
P-SMAD1/5
SMAD1/5
Vinculin
pSMAD/SMAD relative expression
2.5
2.0
1.5
1.0
0.5
0.0
*
**
Ctr FAPs
FOP FAPs
C
SPP1
ActA

Fig. S8

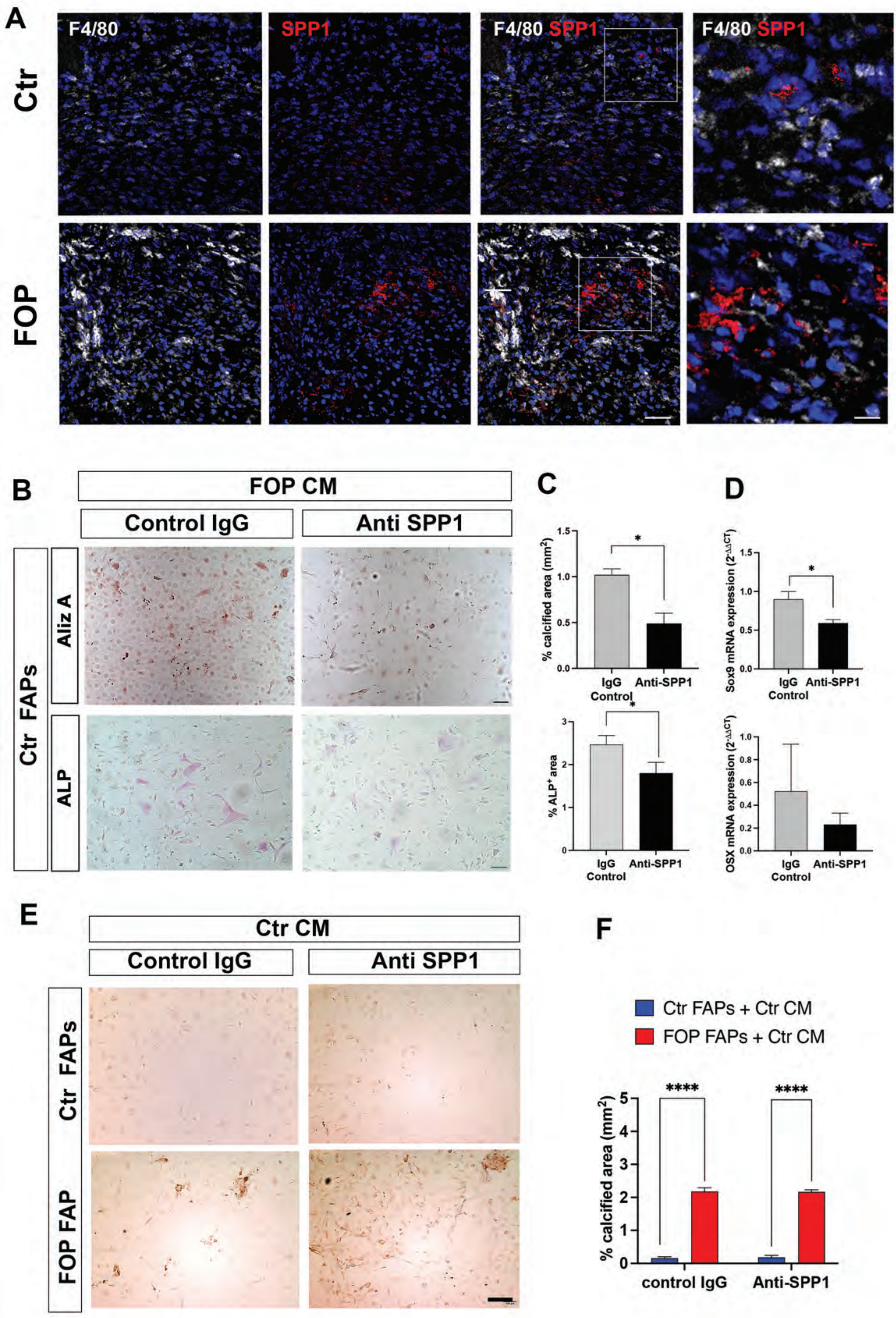
A
Ctr
FOP
F4/80
SPP1
F4/80 SPP1
B
FOP CM
Control IgG
Anti SPP1
Ctr FAPs
Aliz A
ALP
C
% calcified area (mm2)
*
IgG Control
Anti-SPP1
% ALP+ area
D
Sox9 mRNA expression (2-ΔΔCT)
OSX mRNA expression (2-ΔΔCT)
E
Ctr CM
Control IgG
Anti SPP1
Ctr FAPs
FOP FAP
F
Ctr FAPs + Ctr CM
FOP FAPs + Ctr CM
% calcified area (mm2)
****
control IgG
Anti-SPP1

Supplementary Figure Legends

Figure S1 related to Figure 1 "HO in FOP mice and experimental setup of circulating monocytes depletion"

A) μ-CT of Ctr and FOP mice 10, 14, and 21 days after injury of the gastrocnemius by muscle pinch. Scale bar: 5mm

B) Gating strategy and percentage of $CD45^+$ and $CD11b^+$ cells (monocytes) in the blood of CLL *versus* SHAM treated mice, 10 days after injury. Data are presented as mean ± SD (n=3). Statistically significant difference is indicated as * and represents $P < 0.05$ (two-tailed unpaired Student's t-test).

C) Ctr and FOP FAPs treated with conditioned medium derived from Ctr and FOP pro-inflammatory MPs and quantification of mineralized area stained with Alizarin Red. Scale bar: 100 μm. Data are presented as mean ± SD (n=3). Statistically significant differences are indicated as *, ** and *** representing $P < 0.05$, $P < 0.01$ and $P < 0.001$, respectively (two-tailed paired and unpaired Student's t-tests).

Figure S2A related to Figure 2 "Cell clustering in Single-cell RNA sequencing"

A) UMAP embedding of all sequenced samples coloured by individual cluster identities and annotated according to markers' gene expression, based on canonical skeletal muscle gene expression profiles.

B) Heatmap demonstrating cell-type marker gene expression used for meta-cluster classification.

Figure S3 related to Figure 3 "Main upregulated pathways in FOP *versus* Ctr FAPs at 5 and 7 days"

A) DotPlot of significantly upregulated and downregulated genes, sorted by avg_log2[FC] in the comparison FOP *versus* Ctr 5 and 7 days after injury. Dot size indicates the percentage of cells expressing each gene, and dot colour represents the average expression level.

B) Lollipop plots showing upregulated pathways 5 (left panel) and 7 days (right panel) after injury comparing Ctr *versus* FOP conditions in FAPs. The Normalized Enriched Score (NES) indicates the degree of enrichment for each gene set. Positive NES values (in red) represent pathways activated in FOP, while negative NES values (in blue) represent pathways suppressed in FOP. Selected pathways significantly enriched 5 (left panel) and 7 days (right panel) after injury.

Figure S4 related to Figure 4 "Main upregulated pathways in FOP *versus* Ctr MPs at 5 and 7 days"

A) DotPlot of significantly upregulated and downregulated genes, sorted by avg_log2[FC] in the comparison FOP *versus* Ctr 5 and 7 days after injury. Dot size indicates the percentage of cells expressing each gene, and dot colour represents the average expression level

B) Lollipop plots showing upregulated pathways 5 (left panel) and 7 days (right panel) after injury comparing Ctr *versus* FOP conditions in MPs. The Normalized Enriched Score (NES) indicates the degree of enrichment for each gene set. Positive NES values (in red) represent pathways activated in FOP, while negative NES values (in blue) represent pathways suppressed in FOP. Selected significantly pathways at 5 (left panel) and 7 days (right panel).

Figure S5 related to Figure 5 "Enriched pathways in FOP and Ctr mice"

A) Selected pathways enriched in the comparison FOP vs Ctr in Pro-inflammatory macrophages at 5 day (left panel) and 7 days (right panel).
B) Selected pathways enriched in the comparison FOP vs Ctr in Anti-inflammatory macrophages at 5 day (left panel) and 7 days (right panel).
C) Dotplot of selected genes differentially expressed in the comparison FOP vs Ctr in Pro-inflammatory macrophages (annotated in red), anti-inflammatory macrophages (annotated in light-blue) or both (annotated in purple)
D) Activin-A expression in Ctr and FOPs MPs polarized *in vitro*. Gene expression was investigated by rt-qPCR and protein levels were measured by ELISA on cell supernatant. Data are presented as mean ± SD (n=3). Statistically significant differences are indicated as *, ** and *** representing $P < 0.05$, $P < 0.01$ and $P < 0.001$, respectively (two-tailed paired and unpaired Student's t-tests).

Figure S6 related to Figure 5 "*In vitro* polarization of BMDMs isolated from FOP and Ctr mice"
A) Immunofluorescence staining of in vitro polarized BMDMs into pro- (M1) or anti-inflammatory (M2) macrophages using an antibody specific for iNOS (red), nuclei were counterstained with DAPI (blue). Scale bar: 20 µm.
B) Quantification of $iNOS^+$ cells for each condition after polarization. Data are presented as mean ± SD (n=3). Statistically significant differences are indicated as ** and ***, representing $P < 0.01$, and $P < 0.001$ respectively (two-tailed unpaired and paired Student's t-tests).
C) Gene expression levels of *iNos*. Results are normalized to 28S. Data are presented as mean ± SD (n=3). Statistically significant differences are indicated as *, representing $P < 0.05$ (two-tailed unpaired and paired Student's t-tests).
D) Immunofluorescence staining of in vitro polarized BMDMs into pro- (M1) or anti-inflammatory (M2) macrophages using an antibody specific for CD206 (green), nuclei were counterstained with DAPI (blue). Scale bar: 20 µm.
E) Quantification of CD206+ cells, expressed as percentage, for each condition after polarization. Data are presented as mean ± SD (n=3). Statistically significant differences are indicated as *, and **, representing $P < 0.01$, and $P < 0.001$ respectively (two-tailed unpaired and paired Student's t-tests).
F) Gene expression levels of *Cd206*. Results are normalized to 28S. Data are presented as mean ± SD (n=3). Statistically significant differences are indicated as *, representing $P < 0.05$. (two-tailed unpaired and paired Student's t-tests).

Figure S7 related to Figure 6 and 7 "CellChat analysis and signalling pathways"
A) Chord diagrams showing all the significant interactions (Ligand-Receptor pairs) from macrophages to macrophages and FAPs in the 4 condition groups: FOP 5 day (top—left panel), Ctr 5 day (top-right panel), FOP 7 day (bottom—left panel), Ctr 7 day (bottom-right panel).
B) Bar chart of the significantly enriched pathways of FOP and Ctr mice at 5 and 7 days after pinch injury. Pathways colored in blue are significantly enriched in Ctr, and pathways colored in red are significantly enriched in FOP mice.
C) Western blot assay showing the protein expression of pSMAD1/5 (MW = 60 KDa), SMAD 1/5 (MW = 55 KDa) and Vinculin (MW = 124 KDa) in FAPs treated with SPP1 or Activin A for 6 hours.

Data are presented as mean ± SD (n=5). Statistically significant differences are indicated as ∗, and ∗∗, representing P < 0.05, and P < 0.01, (two-tailed paired and unpaired Student's t-test).

Figure S8 related to Figure 8 "SPP1 inhibitions

A) Immunofluorescence staining of gastrocnemius muscle cross-sections of Ctr and FOP mice 7 days post muscle injury using antibodies specific for F4/80 (white) and the SPP1 blocking antibody used for the inhibition experimentis (red). Nuclei were counterstained with DAPI (blue). Scale bar: 20 µm. Scale bar: 8 µm in magnified images inset.

B) Alizarin Red or ALP staining of Ctr FAPs cultured for 6 days in the presence of FOP CM and treated with either anti-SPP1 blocking antibody (2.5 µg/ml) or control IgG. Scale bar: 100 µm.

C) Relative quantification of Alizarine Red$^+$ or ALP$^+$ area of Ctr FAPs cultured for 6 days in the presence of FOP CM and treated with either anti-SPP1 blocking antibody (2.5 µg/ml) or control IgG. Data are presented as mean ± SD (n=3). Statistically significant differences are indicated as *, representing P < 0.05, (two-tailed paired and unpaired Student's t-tests).

D) Gene expression levels of chondro/osteogenic markers Sox9 and Osx in Ctr FAPs cultured for 6 days in the presence of FOP CM and treated with either anti-SPP1 blocking antibody (2.5 µg/ml) or control IgG. Results are normalized to 28S gene used as internal control. Data are presented as mean ± SD (n=3). Statistically significant differences are indicated as * and ** representing P < 0.05, and P < 0.01 respectively (two-tailed paired and unpaired Student's t-tests).

E) Alizarin Red or ALP staining of Ctr and FOP FAPs cultured for 6 days in the presence of FOP CM and treated with either anti-SPP1 blocking antibody (2.5 µg/ml) or control IgG. Scale bar: 100 µm.

F) Quantification of Alizarin Red$^+$ area of Ctr and FOP FAPs cultured for 6 days in the presence of FOP CM and treated with either anti-SPP1 blocking antibody (2.5 µg/ml) or control IgG. Data are presented as mean ± SD (n=3). Statistically significant differences are indicated as **** representing P < 0.0001 (two-tailed unpaired Student's t-test).

Supplementary Tables

Table S1 Expression of marker gene used for population clustering.
Table S2: Deregulated genes in FOP FAPs versus Control FAPs at 5 days.
Table S3: Deregulated genes in FOP FAPs versus Control FAPs at 7 days.
Table S4 Deregulated genes in FOP MPs versus Control MPs at 5 days.
Table S5 Deregulated genes in FOP MPs versus Control MPs at 7 days.
Table S6 Expression of markers gene used for macrophage subpopulation clustering.
Table S7 Deregulated genes in subpopulation of FOP MPs versus Control MPs.
Table S8 Analysis of deregulated cell-cell interactions at 5 days.
Table S9 Analysis of deregulated cell-cell interactions at 7 days.
